# Reconstructing past migratory behaviour of reindeer (*Rangifer tarandus*): Insights from geometric morphometric analysis of proximal phalanx morphology from extant caribou populations

**DOI:** 10.1371/journal.pone.0285487

**Published:** 2023-08-09

**Authors:** Ana Belén Galán López, Maxime Pelletier, Emmanuel Discamps

**Affiliations:** 1 TRACES UMR 5608, CNRS-Université de Toulouse-Jean Jaurès, Toulouse, France; 2 Archaeology, History, Culture and Communication Studies, University of Oulu, Oulu, Finland; New York University, UNITED STATES

## Abstract

Reindeer mobility patterns vary widely in modern ecosystems, notably between more open or more wooded environments. This renders the reconstruction of past reindeer mobility patterns challenging, while being at the same time key if archaeologists want to better understand the role that reindeer herds played in the subsistence and territorial organisation of Prehistoric hunter-gatherer societies. Studying the morphology associated with different habitats and mobility patterns can be a useful method for understanding past reindeer behaviour. To access paleoecological information, the relationship between locomotor anatomy and substrate type can be explored in modern animals and transposed to the past, as previous studies have proven that an animal´s environment affects bone morphology. In this study, 3D Geometric Morphometrics are used to explore the impact of extant reindeer habitat type and mobility pattern on phalanx morphology. Results obtained reflects on the potential archaeological application of such an approach for paleoecological reconstructions. Size and shape of phalanx vary significantly, yet complex to interpret in light of interplaying factors such as subspecies, sexual dimorphism and the influence of migration costs, snow cover and substrate type. If direct application to the archaeological record remains preliminary, this first study permits to highlight promising avenues for future research.

## Introduction

During the Late Pleistocene, the reindeer (*Rangifer tarandus*) was one of the key prey species for many Palaeolithic hunter-gatherer groups [e.g. 1–3]. In Western Europe, several archaeological sequences documented hunting episodes focused on reindeer herds and whose studies have highlighted the central importance of this species in the subsistence of past human groups since the Mousterian to the Magdalenian [e.g. 4–22]. Several authors have suggested that describing the migration patterns of reindeer is crucial for a better understanding of hunter-gatherer groups depending on them, from prehistory to present day in the Arctic [Murray [Unpublished], 23]. Thus, many studies have tried to reconstruct Palaeolithic reindeer migration patterns [14, 20, 24–41]. Research hypotheses varied from following-herd models, sedentary behaviour to migrations carried out on a smaller scale. All, however, pointed out the difficulty of identifying reindeer migration behaviour with the scarcity of investigation tools at hand. This is even more difficult to grasp considering the large ecological plasticity of modern reindeer populations [42, 43].

Extant *Rangifer* (known as caribou in North America, and called reindeer in Eurasia) migrate seasonally to avoid excessive predation on their calving grounds, pests as well as the depletion of local food supplies [44]. Nevertheless, reindeer herds follow different mobility strategies depending on climate conditions, topography and habitat type. In open habitats such us tundra and steppe, mid and long-distance migrations occur [45–48], while animals living in close habitats (e.g. woodlands) tend to move shorter distances [49, 43]. Due to the fact that Pleistocene environments do not necessarily have modern analogues and that reindeer has long been extirpated from Western Europe [7], ethological data can hardly be used to predict past migration patterns, and further palaeoecological data is thus needed. Furthermore, variability in reindeer mobility throughout the Palaeolithic would be expected if one considers the extensive climatic and environmental changes documented for terrestrial ecosystems during the Upper Pleistocene [e.g. 50–52]. For example, with the climate warming at the end of the Late Glacial that lead to reindeer extirpation from southwestern Europe [3, 53], reindeer groups became more fragmented geographically, and different behavioural responses might be expected (e.g. more or less sedentary groups, migration along the Pyrenees, planarly vs. altitudinal migration). Yet, the migration behaviours of reindeer herds during this period are largely unknown, impeding the interpretation of Late Glacial socioeconomic decisions and mobility strategies.

In this study, we seek to develop a new palaeoecological tool for the reconstruction of reindeer mobility, taking their bone remains into account in regards to the archaeological record. Since locomotor adaptations are closely related to habitat preferences and mobility patterns [39–41, 54–58], behaviour can be inferred from the study of animal morphology. Ecomorphological research has proposed a relationship between the degree of mobility (or habitat preferences) and the functional morphology of limb bones for a wide range of taxa [54, 56, 58–67], and thus made it possible to successfully reconstruct their habitat [e.g. 54, 56, 58, 60, 61, 63–66].

In this regard, caribou’s concave hooves are an interesting adaptation to the harsh and treacherous conditions of northern environments. Their feet are the interface between the environmental substrate and their bodies, and as migratory animals, they rely on their hooves to carry them long distances [68]. In deep snow, caribou walk on their toes, which act like snowshoes. In the fall, their feet begin to harden, and they develop particularly sharp edges that can easily break through ice layers to find food. Likewise, their feet can be used as paddles for swimming or as ice picks when navigating steep, rocky, and icy mountain slopes. The large and flexible feet of caribou also make them extremely useful on uneven, spongy ground when it is not snowing [69]. Furthermore, phalanges are rather dense and strong and are often preserved in archaeological sites.

Due to its use both in livestock practices and domestication, reindeer has been a subsistence resource for many past and contemporary populations. Thus, several studies on the Fennoscandian subspecies (*Rangifer tarandus tarandus* and *R*. *t*. *fennicus*) have been carried out in relation to its postcranial morphology (e.g. cross-sectional morphology, entheseal changes) and identification in the archaeological record (particularly to distinguish the different stages of its domestication). They have provided an important understanding of this subject, which is key in reconstructing the mobility and lifestyles of northern peoples [68, 70–77]. In parallel, the increased development of geometric morphometrics (GMM) provides a powerful set of techniques for distinguishing subtle morphological differences among members of the same species. It was successfully applied to the study of ecomorphological patterns in several instances [67, 75–80].

Building from these previous works, we here extend the application of 3D GMM to phalanges and to a larger sample of wild sedentary and (latitudinal or altitudinal) migratory populations, in order to explore the potential link between proximal phalanges morphology and reindeer mobility and habitat. The choice of the proximal phalanx is due to the fact that it is a often element in the archaeological record of the potential sites to be studied.

Thus, the main purpose of this work is to determine if there are size and shape differences in proximal phalanx morphology between subspecies (*Rangifer tarandus caribou*, *Rangifer tarandus granti*, *Rangifer tarandus groenlandicus*, *Rangifer tarandus pearyi*) that have different mobility patterns (altitudinal, longitudinal, sedentary) and live in different habitats (mountain, boreal forest and tundra). Moreover, since *Rangifer tarandus caribou* subspecies lives in mountainous areas, boreal forests, tundra, and undertakes altitudinal and longitudinal migrations or has a sedentary behaviour [43, 81], this subspecies (see Sampling section for further details) will also be studied separately.

Although morphological studies of reindeer often consider sex a key variable [75, 76, 82] or even other ungulate osteometric studies [83, 84], the presence of sexual dimorphism will be just visually explored in size (distribution of the log-centroid sizes) in the subspecies *Rangifer tarandus caribou* for which there is a limited but available sample of males and females.

Finally, as previous studies have shown that allometry is significant [77], this study will explore the effect of size on the shape of proximal phalanges of both forelimbs and hindlimbs.

## Material and methods

### Sampling extant caribou populations

**Fig 1:**
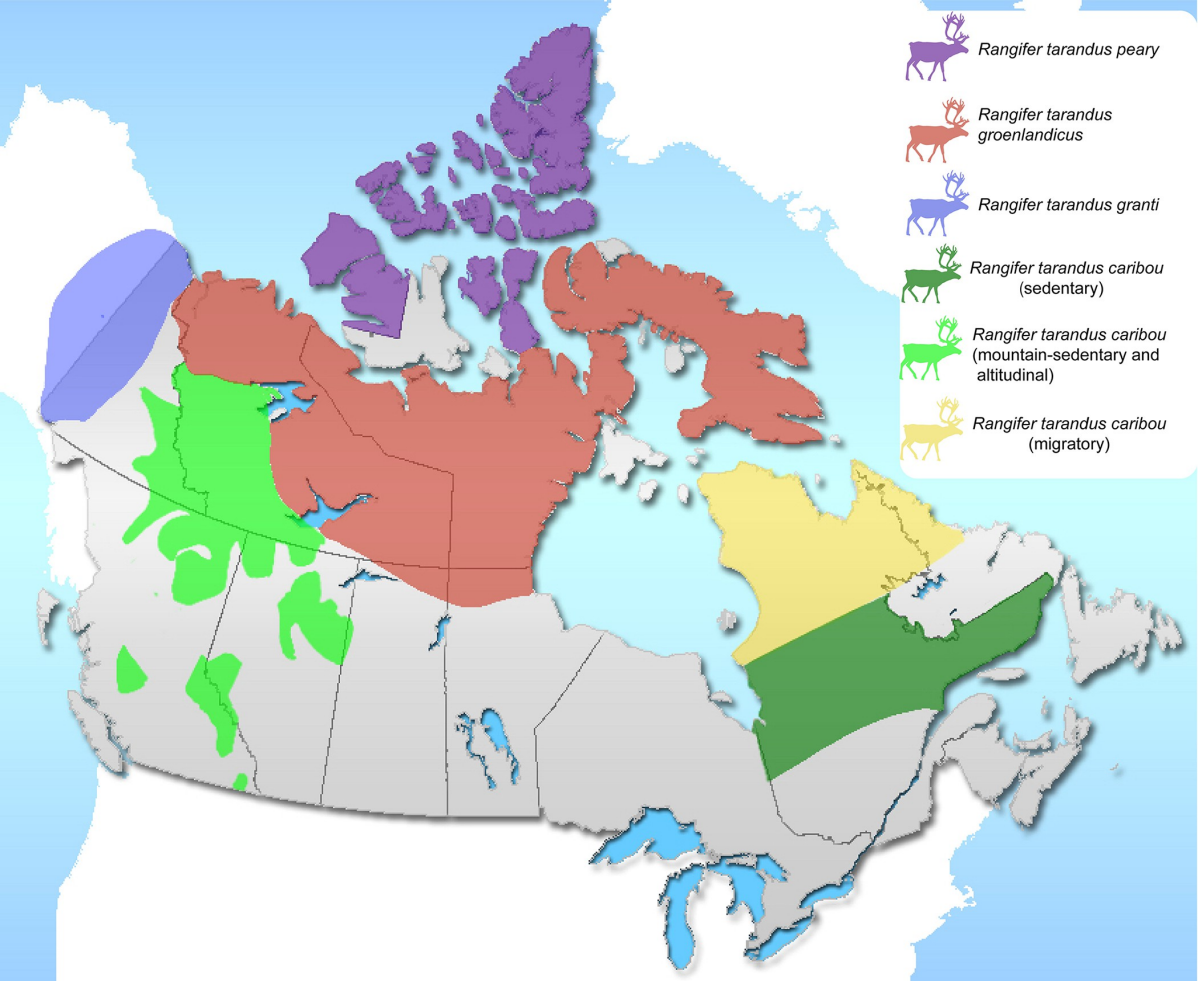
Location of the *Rangifer tarandus* subspecies in North America. Origin of the specimens used in the present study (notice that this distribution area partially coincides with the current *Rangifer tarandus* distribution in North America, which is wider). Map base by Qyd, Wikimedia Commons, CC Public domain, https://commons.wikimedia.org/wiki/File:Canada-provinces_layout.png.

*Rangifer tarandus* has a wide circumpolar distribution. For this study, we acquired proximal phalanges of individuals belonging to the four subspecies of North American caribou (see Fig 1) (mainly from Canada). These subspecies occupy different habitat settings (boreal, montane and arctic environments), sometimes overlapping, and adopt different mobility strategies. Across this large area, caribou exhibit a wide variation in ecology, genetics, behaviour and morphology. Biologists generally distinguish two main mobility patterns for caribou: migratory and sedentary [42]. However, in mountainous settings some caribou populations perform these migratory movements altitudinally [85], calling it altitudinal or elevational migration.

The subspecies selected for this study are the following:

1) *R*. *t*. *caribou* (which includes Eastern caribou/Migratory woodland caribou, mountain caribou and boreal caribou/woodland caribou) inhabits different environments and performs short, medium and long-distance movements. In Quebec, Woodland caribou living south of 55° N are forest-dwellers and seasonally move relatively short distances compared to their tundra counterparts [86, 87], between 50 and 150 kilometres depending on the herd [49, 48, 88, 89]. Eastern or Migratory Woodland caribou group two big herds, George River and Leaf River, that, despite belonging to Woodland caribou, behave like Barren-Ground or tundra caribou, performing migration on long distances ranging between 1120–1770 kilometres on average per year [90], from open tundra settings to boreal forest habitats [91]. Mountain caribou inhabits mountain and boreal settings. Some herds perform altitudinal migrations, with overall distances between 140–240 kilometres only in spring migration, depending upon the location of their calving areas, while others remain relatively sedentary, travelling shorter distances under 100 kilometres [81, 92].

2) The Peary caribou (*R*. *t*. *pearyi*) inhabits the treeless arctic tundra [44, 93–95] in the Arctic Archipelago of Canada (except Baffin Island). These insular caribou notably differ from other tundra populations, mostly by their size (smallest of all *Rangifer tarandus* subspecies). Each season, they perform migrations between islands, by crossing the sea ice and/or swimming [43, 45, 96–98] covering migration distances of up to 500 kilometres [45, 93]. As an example, it has been estimated that seasonal migrations within the Prince of Wales-Somerset-Boothia complex can cover between 300 to 500 kilometres, if not more [99].

3 & 4) The Grant’s caribou (*R*. *t*. *granti*) and the barren-ground caribou (*R*. *t*. *groenlandicus*), referred as Barren-Ground caribou, are alternatively considered to be one or two subspecies [69]. The first one is mainly found in Alaska and its adjacent Canadian territories, the second one in Canada only (Nunavut, Northwest Territories). These tundra caribou include some of the largest herds in North America, numbering hundreds of thousands of individuals that make very long-distance migrations, ranging between 800 and 5055 kilometres (annual migration) depending on the herds and the year [81, 100–103]. Their habitats are mainly prostrate dwarf shrub tundra and upright shrub tundra [94].

For this research we compiled a sample of 34 fore- and 44 hindlimb proximal (first) phalanges belonging to the four subspecies of caribou described above (Tables 1 and 2). All of them were adults and specimens with bone pathologies were excluded from the study. Samples were obtained from biologists in the Ministry of Forests, Lands, Natural Resource Operations and Rural Development, British Columbia, who provided us with mountain caribou samples and related information about herds (type of herd, sex if known and location). Peary caribou, Barren-Ground and Grant’s caribou specimens were collected from the Canadian Museum of Nature (Ottawa), which owns one of the largest collections of caribou in the country. Eastern migratory and short distance woodland caribou were obtained with the help of Biologists from the Forest and Wildlife branch of the Québec Government, the Prehistory and bioarchaeology lab from Laval University and the archaeozoology laboratories of the University of Montréal.

**Table 1:** Sample used in the present study.

(a)				
**Forelimb Phalanges**	**Altitudinal**	**Planarly**	**Sedentary**	**Total**
***Rangifer tarandus caribou***				
Mountain	6		5	**11**
Tundra		6		**6**
Boreal Forest			7	**7**
***Rangifer tarandus ganti***				
Tundra		4		**4**
***Rangifer tarandus groenlandicus***				
Tundra		2		**2**
***Rangifer tarandus pearyi***				
Tundra		4		**4**
**Total**	**6**	**16**	**12**	**34**
(b)				
**Hindlimb Phalanges**	**Altitudinal**	**Planarly**	**Sedentary**	**Total**
***Rangifer tarandus caribou***				
Mountain	12		4	**16**
Tundra		10		**10**
Boreal Forest			10	**10**
***Rangifer tarandus ganti***				
Tundra		2		**2**
***Rangifer tarandus groenlandicus***				
Tundra		2		**2**
***Rangifer tarandus pearyi***				
Tundra		4		**4**
**Total**	**12**	**18**	**14**	**44**

List of the fore-(a) and hindlimb (b) phalanges according to subspecies (*R*.*t*. *caribou*, *R*.*t*. *granti*, *R*.*t*. *groenlandicus* and *R*.*t*. *pearyi*), habitat (mountain, tundra and boreal forest) and mobility type (sedentary, planarly and altitudinal).

**Table 2:** Detailed sample.

**Forelimb Phalanx**	**Male**	**Female**	**Unknown**	**Total**
***Rangifer tarandus caribou***	4	3	17	**24**
***Rangifer tarandus granti***	4	0	0	**4**
***Rangifer tarandus groenlandicus***	0	0	2	**2**
***Rangifer tarandus pearyi***	3	0	1	**4**
**Total**	**11**	**3**	**20**	**34**
**Hindlimb Phalanx**	***Male***	***Female***	***Unknown***	***Total***
***Rangifer tarandus caribou***	3	7	26	**36**
***Rangifer tarandus granti***	2	0	0	**2**
***Rangifer tarandus groenlandicus***	2	0	0	**2**
***Rangifer tarandus pearyi***	4	0	0	**4**
***Total***	**11**	**7**	**26**	**44**

Detailed of the fore- and hindlimb phalanges according to subspecies (*R*.*t*. *caribou*, *R*.*t*. *granti*, *R*.*t*. *groenlandicus* and *R*.*t*. *pearyi*) and sex (male, female, unknown).

To facilitate data analysis, each individual was given a unique number (from DPpha 1 to DPpha X, with DP standing for “DeerPal”, the acronym of the project). A detailed table listing the habitat and mobility pattern of each phalanx, the number of our unique ID, as well as population information is provided in the S1 Table.

The fact that sedentary populations are classified as “endangered” by Canadian Government makes it more difficult to obtain samples from these herds (since there are very few specimens in museums, the only way to obtain them was through samples sent by biologists when a caribou was killed in an accident or found dead) resulting in uneven samples. Unfortunately, sex was unknown for almost all individuals (n = 14 forelimb phalanges; n = 18 hindlimb phalanges).

Groups were considered according to Subspecies (*Rangifer tarandus caribou*, *Rangifer tarandus granti*, *Rangifer tarandus groenlandicus*, *Rangifer tarandus pearyi*), Mobility (Altitudinal for populations migrating vertically, Planarly for populations migrating mostly on a latitudinal or longitudinal plane, and Sedentary) and Habitat (Mountain, Boreal Forest and Tundra) (see Table 1). In order to analyse *R*.*t*. *caribou* subspecies, specimens were grouped according to habitat and mobility pattern (see Table 3).

**Table 3:** Acronyms used in the present study.

Name	Acronym
Rtcaribou_BorealForest_Sedentary	Rtcaribou_BForest_Sed
Rtcaribou_Mountain_Altitudinal	Rtcaribou_Mount_Alt
Rtcaribou_Mountain_Sedentary	Rtcaribou_Mount_Sed
Rtcaribou_Tundra_Planarly	Rtcaribou_Tundra_Plan
Rtgranti_Tundra_Planarly	Rtgranti_Tundra_Plan
Rtgroenlandicus_Tundra_Planarly	Rtgroenlandicus_Tundra_Plan
Rtpeary_Tundra_Planarly	Rtpeary_Tundra_Plan

List of acronyms used in the present study according to subspecies (*R*.*t*. *caribou*, *R*.*t*. *granti*, *R*.*t*. *groenlandicus* and *R*.*t*. *pearyi*), habitat (mountain, tundra and boreal forest) and mobility type (sedentary, planarly and altitudinal).

## Methods

### Data acquisition and 3D geometric morphometrics

Phalanges were individually scanned using a NextEngine 3D laser Scanner (at the Anthropology Department of the Université de Montréal, Canada), in order to obtain a highly accurate 3D surface model of each bone. All the 3D models are openly available on NAKALA (https://doi.org/10.34847/nkl.fa00p31c).

Geometric morphometrics (GMM) is a quantitative approach which allows the comparison of bone shapes and the visualization of significant morphological differences between groups of specimens while retaining the element of shape information related to size [104, 105].

Because of the uncertainty in terms of the anatomical position of the curve and surface semilandmarks, sliding step was carried out in curves along their tangent vectors and surface points within their tangent planes in order to minimize the bending energy of the landmark configurations and the template during the Thin-Plate Spline (TPSE) interpolation [106, 107]. After that, the resulting landmark configurations were rotated, translated and scaled using the Generalized Procrustes Analysis (GPA) [108, 109]. This analysis eliminates the effect of position and orientation of the configurations, and limits the effect of size [110–112], yielding Procrustes shape coordinates that were subsequently analysed.

**Fig 2:**
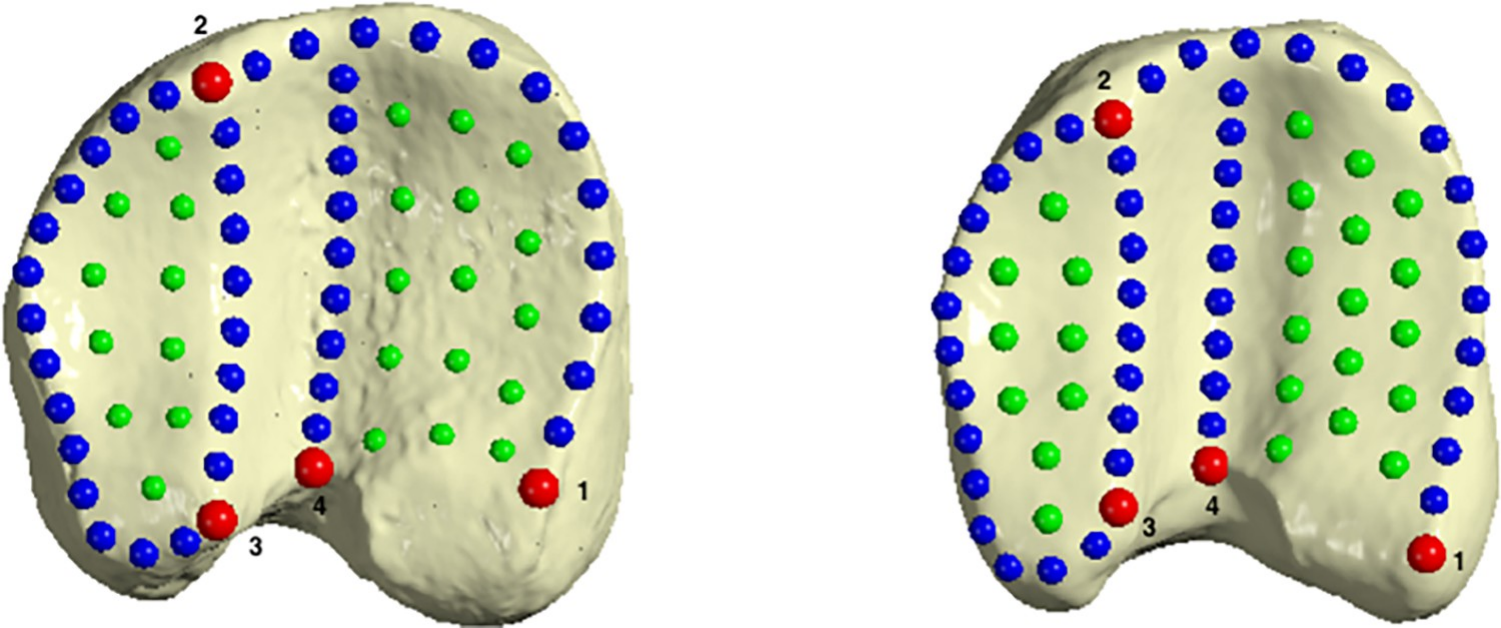
Landmarks and semilandmarks location. Location of anatomical landmarks (red), curve semilandmarks (blue) and surface semilandmarks (green) placed on proximal epiphyses for fore- (left) and hindlimb (right) first phalanges. The definitions of landmarks are given in Tables 4 and 5.

Considering that proximal phalanges are often found fragmented in Palaeolithic sites, we focused our work on proximal epiphyses, in order to allow future applications to a larger part of the archaeological record. The round shape of articular surfaces of these phalanges make it difficult to define true anatomical landmarks, and thus semilandmarks were preferred to define the complex morphology of the joint [106]. We placed landmarks and semilandmarks on areas not affected by entheseal changes and paleopathologies [70, 72]. Two landmark configurationtemplates were made with a total of 4 landmarks (LM), 43 curve semilandmarks (CSLM) and 25 surface semilandmarks (SSLM) for forelimb phalanges and 4 LM, 45 CSLM and 25 SSLM for hindlimb phalanges, that were digitized to get the form of the proximal epiphyses (Fig 2, Tables 4 and 5). Digitalization was carried out using ViewBox 4.1.0.12 (dHAL software, Kifissia, Greece).

**Table 4:** List of anatomical landmarks and semilandmarks (forelimb phalanx).

Landmark	Description
1	Abaxial facet of the proximal phalanx, on the edge of the intersection of the curve, just above the small medial articular facet. Insertion tuber edge.
2	Most antero-medial point of the lateral glenoid cavity
3	Most postero-medial point of the lateral glenoid cavity
4	Most postero-distal point of the sagittal groove
**Curves (CSLM)**	
5:16	Crest between LM 1 and LM 2.
17:30	Crest between LM 2 and LM 3.
31:38	Crest between LM 3 and LM 2.
39:47	Crest between LM 39 and CSLM 14.
**Surfaces (SSLM)**	
48:57	Lateral articular surface patch.
58:72	Medial articular surface patch.

Definition of anatomical landmarks and semilandmarks for forelimb proximal phalanx shown in Fig 2.

**Table 5:** List of anatomical landmarks and semilandmarks (hindlimb phalanx).

Landmark	Description
1	Abaxial facet of the proximal phalanx, on the edge of the intersection of the curve, just above the small medial articular facet. Insertion tuber edge.
2	Most antero-medial point of the lateral glenoid cavity
3	Most postero-medial point of the lateral glenoid cavity
4	Most postero-distal point of the sagittal groove
**Curves (CSLM)**	
5:18	Crest between LM 1 and LM 2.
19:32	Crest between LM 2 and LM 3.
33:40	Crest between LM 3 and LM 2.
41:49	Crest between LM 4 and CSLM 16.
**Surfaces (SSLM)**	
50:58	Lateral articular surface patch.
59:74	Medial articular surface patch.

Definition of anatomical landmarks and semilandmarks for hindlimb proximal phalanx shown in Fig 2.

### Statistical analyses: Size and shape differences

First, the four subspecies were analysed altogether. Then, since *R*. *t*. *caribou* subspecies constituted a greater percentage (70.5%-forelimb phalanges and 81.8%-hindlimb phalanges) of the sample, the same analyses were only conducted on individuals from this subspecies.

Thus, based on log-transformed centroid sizes for forelimb and hindlimb phalanges, the size differences were assessed using Kruskal-Wallis tests (threshold set at p< 0.05) for the specimens pooled by “Subspecies”, “Habitat”, “Mobility”, and the interactions “Subspecies+Habitat”, “Subspecies+Mobility” and “Mobility+Habitat”. Then, multiple Wilcoxon-rank tests were used to compare these groups according to these categories. P values were corrected using the “Benjamini-Hochberg” method to decrease the false discovery rate [113].

A Principal Component Analysis (PCA) based on Procrustes Coordinates was conducted to observe shape variation in the morphospace. Using a Thin-Plate Spline (TPS) interpolation function [108] we generated a 3D digital mesh for each articular surface respectively that was wrapped toward the Procrustes grand mean to understand variations along the principal axes. From the surface of the Procrustes mean configurations [114], shapes at the extremes of the axes of the principal components were visualized and magnified by a scale factor of 0.1 (in order to improve the visualization of the morphological differences), using the function “tps3d” in the “Morpho” package [115].

A multivariate analysis of variance (MANOVA) on PC scores was used to estimate shape differences between these groups. Additionally, in order to test the assignment accuracy for each category, a Canonical Variate Analysis (CVA) was computed on PC scores (data dimensionality reduction) by calculating the cross-validated correct classification and the Kappa metric (see S1 File). We avoided overfitting [116] by never exceeding 95% of the variance cumulative and by never using more PCs than the number of specimens from the smallest group or category.

As sex variable was known in some specimens from *R*. *t*. *caribou* subspecies, sexual size dimorphism was only explored in this subspecies through the Kruskal-Wallis test and if significant, then multiple Wilcoxon-rank test. We also examined shape sexual dimorphism using Principal Component Analysis, Thin-Plate-Spline, and a MANOVA test, as described previously.

Multivariate regressions of shape variables on log-transformed centroid sizes were used to analyse allometry.

All these analyses were performed on RStudio v.4.1.1 [117] using “geomorph” [118] and “Morpho” packages [115].

## Results

### Size variation considering the four subspecies altogether

#### Forelimb proximal phalanx

**Fig 3:**
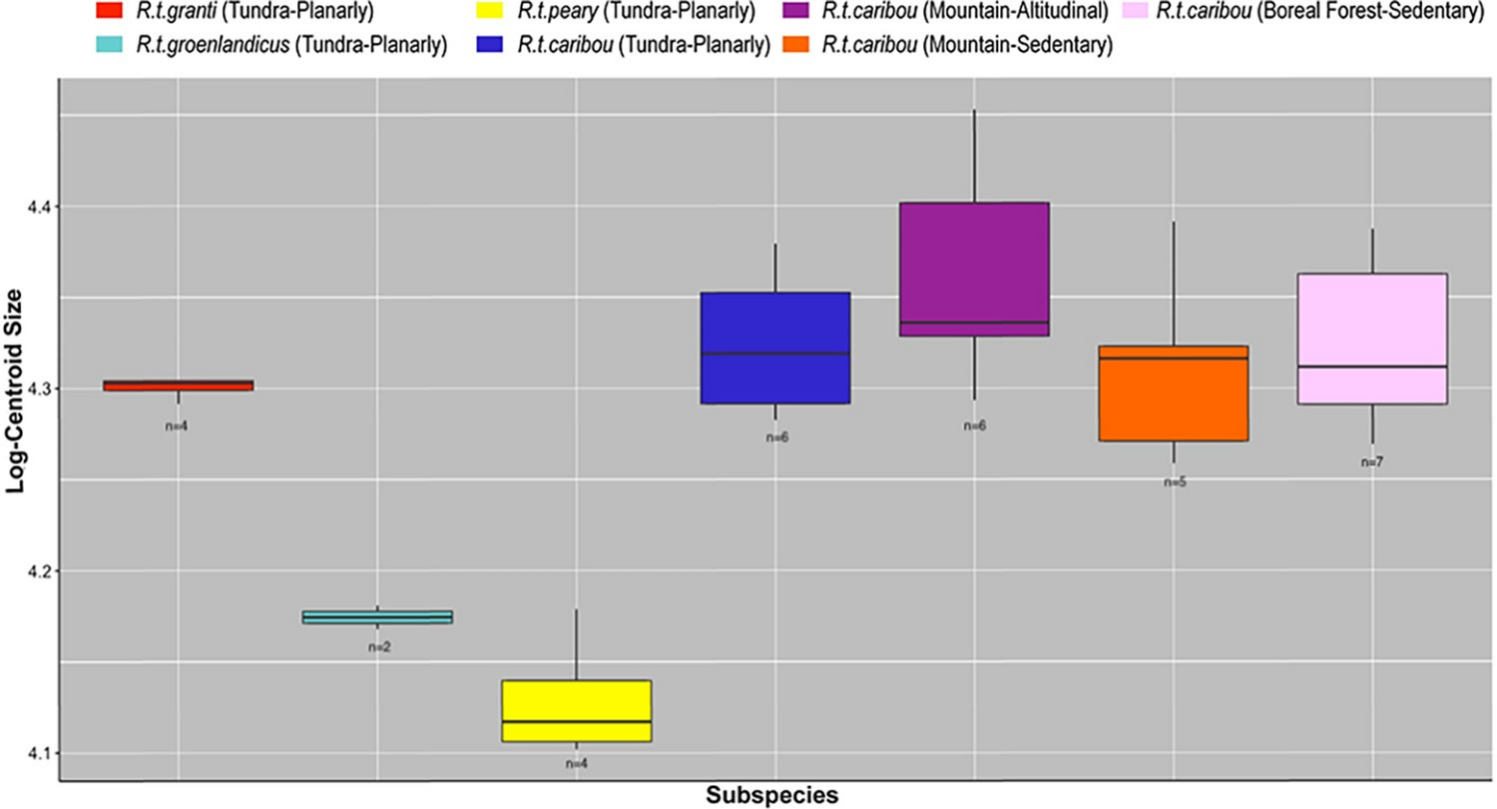
Boxplots showing the size variation. Log-transformed centroid size boxplot distribution on forelimb phalanges according to subspecies (*R*.*t*. *caribou*, *R*.*t*. *granti*, *R*.*t*. *groenlandicus* and *R*.*t*. *pearyi*), habitat (mountain, tundra and boreal forest) and mobility type (sedentary, planarly and altitudinal).

Kruskal-Wallis tests show significant size differences in almost all of the variables: subspecies (p<0.002), habitat (p<0.05), mobility (p<0.05), and most of the interactions between these variables (Table 6). Mobility+Habitat interaction did not yield significant differences. When subspecies category is included in the interaction for both mobility and habitat, there are always significant differences (p<0.05). However, it must be noted that, for all subspecies expect *R*.*t*. *caribou*, only one type of habitat (tundra) and mobility (planarly) are represented in our sample, a composition that affects the statistical results obtained by Kruskall-Wallis tests. Fig 3 highlights the fact that size differences are mainly due to the subspecies variable (i.e. *R*.*t*. *groenlandicus* and *R*.*t*. *peary* are smaller).

**Table 6:** Kruskal-Wallis results.

	Df	Chi-square	P value
**Forelimb Proximal Phalanx**			
Subspecies	3	15.132	**0.0017**
Habitat	2	6.1572	**0.046**
Mobility	2	7.4951	**0.0235**
Subspecies*Habitat	5	15.361	**0.0089**
Subspecies*Mobility	5	16.699	**0.0051**
Mobility*Habitat	3	7.623	0.054
**Hindlimb Proximal Phalanx**			
Subspecies	3	14.235	**0.0026**
Habitat	2	7.0519	**0.0294**
Mobility	2	8.9936	**0.0111**
Subspecies*Habitat	5	14.992	**0.0104**
Subspecies*Mobility	5	16.933	**0.0046**
Mobility*Habitat	3	9.67	**0.0215**

Results of the Kruskal-Wallis tests on log-transformed centroid size for fore and hindlimb phalanges according to subspecies (*R*.*t*. *caribou*, *R*.*t*. *granti*, *R*.*t*. *groenlandicus* and *R*.*t*. *pearyi*), habitat (mountain, tundra and boreal forest) and mobility type (sedentary, planarly and altitudinal) and their interactions. A significant contribution was considered for *P* value <0.05 **(in bold**).

Pairwise comparisons did not show size differences when they were displayed by subspecies+mobility+habitat. However, although it is not statistically significant, larger sizes correspond to male specimens which performs altitudinal migrations (*R*. *t*. *caribou*, mountain setting) (Table 7) as it can be observed in boxplots (Fig 3).

**Table 7:** Pairwise comparisons of log-transformed centroid-sizes.

*Subspecies*	*Rtcaribou_BorealForest_Sedentary*	*Rtcaribou_Mountain_Altitudinal*	*Rtcaribou_Mountain_Sedentary*	*Rtcaribou_Tundra_Planarly*	*Rtgranti_Tundra_Planarly*	*Rtgroenlandicus_Tundra_Planarly*
*Rtcaribou_Mountain_Altitudinal*	0.530	-	-	-	-	-
*Rtcaribou_Mountain_Sedentary*	0.943	0.211	-	-	-	-
*Rtcaribou_Tundra_Planarly*	0.943	0.660	0.801	-	-	-
*Rtgranti_Tundra_Planarly*	0.801	0.161	0.831	0.943	-	-
*Rtgroenlandicus_Tundra_Planarly*	0.161	0.161	0.171	0.161	0.191	-
*Rtpeary_Tundra_Planarly*	0.099	0.099	0.105	0.099	0.123	0.399

Pairwise comparisons of log-transformed centroid sized for forelimb phalanx between the different groups according to subspecies (*R*.*t*. *caribou*, *R*.*t*. *granti*, *R*.*t*. *groenlandicus* and *R*.*t*. *pearyi*), habitat (mountain, tundra and boreal forest) and mobility type (sedentary, planarly and altitudinal) using Wilcoxon rank sum test after “Benjamini-Hochberg” correction. A significant contribution was considered for *P* value <0.05 (**in bold**).

#### Hindlimb proximal phalanx

**Fig 4:**
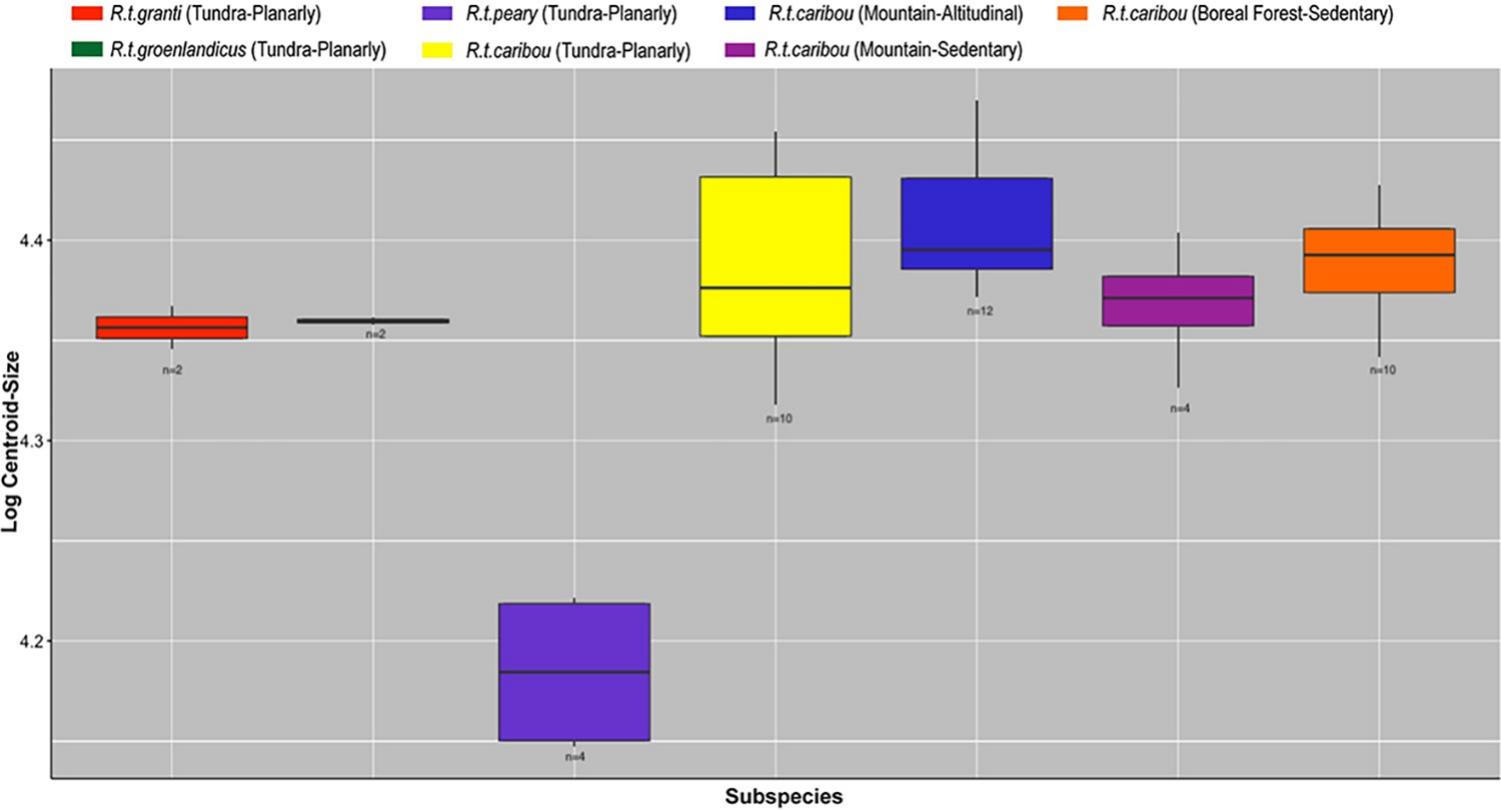
Boxplots showing the size variation. Log-transformed centroid size boxplot distribution on hindlimb phalanges according to subspecies (*R*.*t*. *caribou*, *R*.*t*. *granti*, *R*.*t*. *groenlandicus* and *R*.*t*. *pearyi*), habitat (mountain, tundra and boreal forest) and mobility type (sedentary, planarly and altitudinal).

Kruskal-Wallis´results (see Table 6) on log-transformed centroid sizes yielded significant differences (p<0.05) among all the categories: Subspecies, Habitat, Mobility, Subspecies+Mobility, Subspecies+Habitat and Mobility+Habitat. Yet, again, this statistical test is affected by our sample composition. Pairwise comparisons (Table 8) by subspecies+mobility+habitat showed size differences (p<0.05) between *R*.*t*. *peary* and three groups of *R*.*t*. *caribou* (BForest_Sedentary; Mountain_Altitudinal and Tundra_Planarly), the former being smaller (Fig 4).

**Table 8:** Pairwise comparisons of log-transformed centroid-sizes.

*Subspecies*	*Rtcaribou_BForest_Sedentary*	*Rtcaribou_Mountain_Altitudinal*	*Rtcaribou_Mountain_Sedentary*	*Rtcaribou_Tundra_Planarly*	*Rtgranti_Tundra_Planarly*	*Rtgroen_Tundra_Planarly*
*Rtcaribou_Mountain_Altitudinal*	0.460	-	-	-	-	-
*Rtcaribou_Mountain_Sedentary*	0.460	0.180	-	-	-	-
*Rtcaribou_Tundra_Planarly*	1	0.415	0.826	-	-	-
*Rtgranti_Tundra_Planarly*	0.415	0.125	0.602	0.602	-	-
*Rtgroen_Tundra_Planarly*	0.415	0.125	0.602	0.826	1	-
*Rtpeary_Tundra_Planarly*	**0.041**	**0.041**	0.125	**0.041**	0.245	0.245

Pairwise comparisons of log-transformed centroid sized for hindlimb phalanx between the different groups according to subspecies *(R*.*t*. *caribou*, *R*.*t*. *granti*, *R*.*t*. *groenlandicus* and *R*.*t*. *pearyi*), habitat (mountain, tundra and boreal forest) and mobility type (sedentary, planarly and altitudinal) using Wilcoxon rank sum test after “Benjamini-Hochberg” correction. A significant contribution was considered for *P* value <0.05 (**in bold**).

### Size variation within *Rangifer tarandus caribou*

*R*. *t*. *caribou* is the only subspecies in our sample that include specimens of varying habitats and mobility. Kruskal-Wallis yielded no statistical differences (Table 9) on size for *Rangifer tarandus caribou* subspecies. Therefore, no pairwise comparisons were carried out. A very small size variation is perceptible in the boxplots (see S2 File) when both habitat and mobility are considered, even if specimens that carried out altitudinal migration have slightly bigger phalanges. These small differences could be explained, at least in part, by differences in sex ratios between subpopulations (see Table 2), although this would be difficult to explore without a larger sample size.

**Table 9:** Kruskal-Wallis results on log-transformed centroid size.

	*Df*	*Chi-square*	*P value*
**Forelimb Proximal Phalanx**			
Habitat	2	0.19013	0.9093
Mobility	2	2.04	0.3606
Mobility *Habitat	3	2.2869	0.515
**Hindlimb Proximal Phalanx**			
Habitat	2	0.62883	0.7302
Mobility	2	2.9569	0.228
Mobility *Habitat	3	3.8991	0.2726

Results of the Kruskal-Wallis tests on log-transformed centroid size for fore and hindlimb phalanges for *Rangifer tarandus caribou* subspecies according to habitat (mountain, tundra and boreal forest) and mobility type (sedentary, planarly and altitudinal) and their interaction. A significant contribution was considered for *P* value <0.05 (**in bold**).

### Shape variation results considering the four subspecies altogether

#### Forelimb proximal phalanx

**Fig 5:**
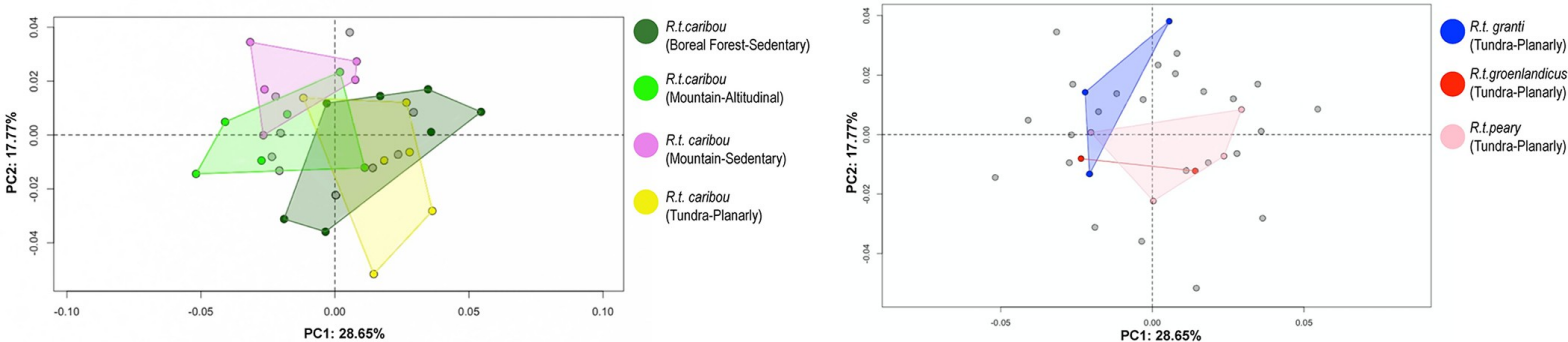
Principal component analysis. Scatter plots of the two first axes (PC1 and PC2) of principal component analyses performed on the shape data (forelimb phalanx) according to subspecies (*R*.*t*. *caribou*, *R*.*t*. *granti*, *R*.*t*. *groenlandicus* and *R*.*t*. *pearyi*), habitat (mountain, tundra and boreal forest) and mobility type (sedentary, planarly and altitudinal). The result of the same PCA is expressed in two scatterplots (for a better visualization). On the left, the highlighted individuals correspond to *R*.*t*. *caribou* subspecies, and on the right, those of the remaining subspecies.

The first 2 PCs explain 46.42% of the morphological variance (Fig 5). Shape variation along PC1 accounted for 28.65% of the variance while PC2, 17.77%. Although there are overlaps according to both Habitat and Mobility type (Sedentary, Planarly and Altitudinal), specimens whose migration is altitudinal showed their variation mainly along negative values of PC1. No pattern is evident for sedentary individuals, with Rtcaribou_BForest_Sed falling mainly towards positive PC1 values and negative PC2, while Rtcaribou_Mount_Sed are on negative PC1 and positive PC2 values. Planarly migraters are spread across the morphospace: Rtcaribou_Tundra_Plan are distributed along PC1 positive and PC2 negative values, Rtpeary_Tundra_Plan at the center of the morphospace, whereas Rtgranti_Tundra_Plan and Rtgroenlandicus_Tundra_Plan are mainly towards negative values of PC1.

**Fig 6:**
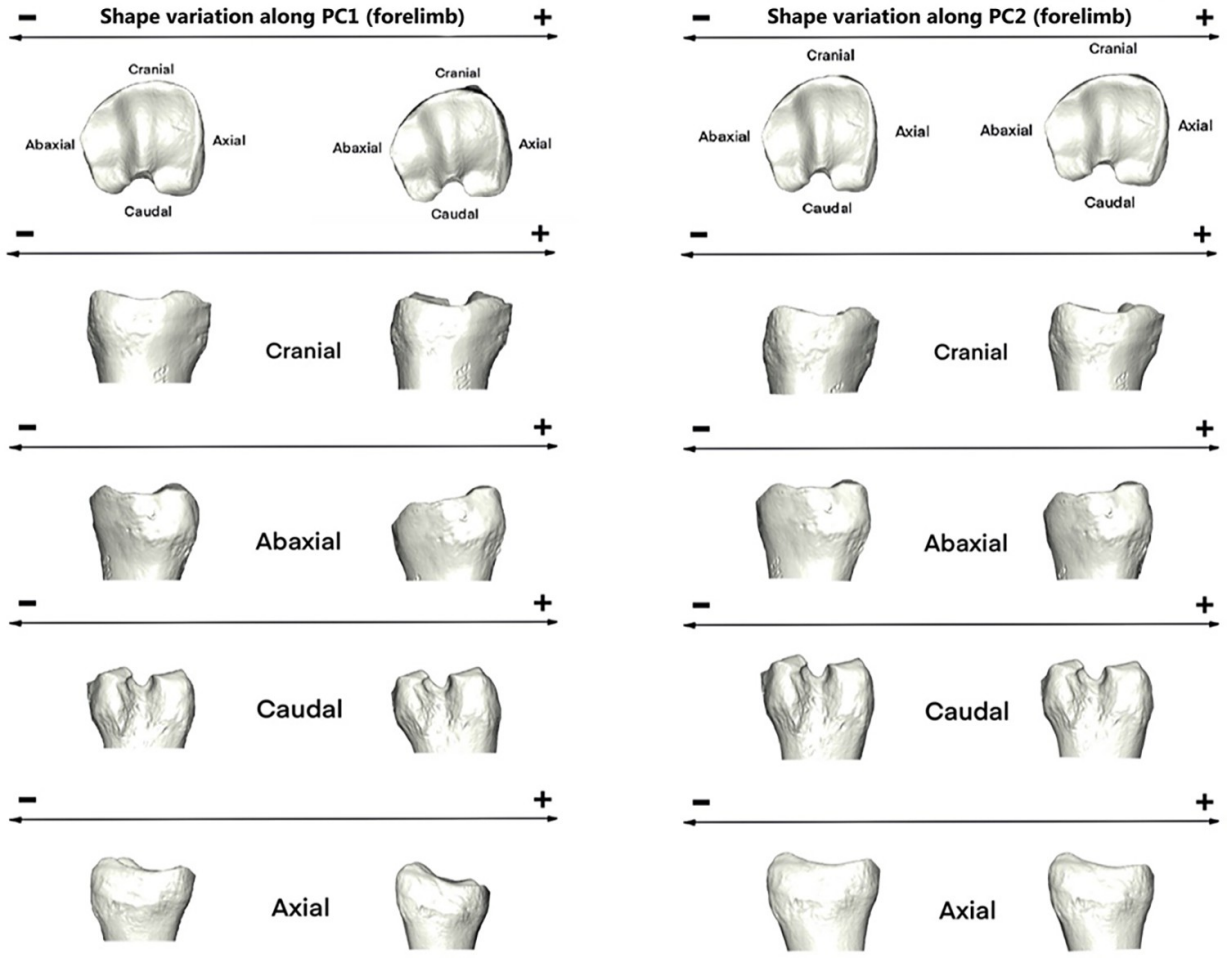
Visualization of shape variation. Shape variation visualized by deforming the mean shape along negative and positive PC1 and PC2 values (magnified by a scale factor of 0.1). Forelimb phalanges.

Towards the positive values of PC1, proximal morphology is more massive towards cranial side and mediolaterally narrower (Fig 6). Axial ridge resulted to be straighter and abaxial articular surface broader. However, towards negative values, proximal joint appeared to be more prominent mediolaterally than craniocaudally. Middle groove was deeper towards the negative values.

Thus, the abaxial view is more massive towards the positive values of PC1 (mainly individuals from tundra habitat and planarly migration) and narrower towards the negative values of the same axis (mostly Rtcaribou_Mountain_Altitudinal specimens). In the caudal view, the morphology tends to be more inwardly curved towards positive values of PC1 and straighter towards negative ones. In the same way, in the axial view, a greater protuberance is observed in the lower part of the articular surfaces towards the positive values of the PC1 axis and a flatter configuration towards the negative ones.

Regarding PC2, the main differences are observed in the proximal view of the articular surface. In the axial view, a narrower configuration is observed towards the positive values of this axis. The theoretical shape at the PC2 minimum showed a proximal joint that is larger craniocaudally and narrower on an axial-abaxial axe and the opposite it is shown towards its maximum shape and occurs more often on mountain specimens.

MANOVA results (Table 10) produced significant differences in shape according to Habitat (p = 0.0048); Ssp+Habitat+Mobility (p = 0.0005); Subspecies*Habitat (p = 0.0004); Subspecies*Mobility (p = 0.0065) and Mobility*Habitat (p = 0.0083). Contrary, there is no shape difference according to Subspecies and Mobility. When analyses are displayed by pooling according to Ssp+Habitat+Mobility significant differences were found between: on one hand, Rtcaribou_BForest_Sed, and, on the other hand, Rtcaribou_Tundra_Plan (p = 0.016) and Rtpeary_Tundra_Plan (p = 0.025) (see Table 11).

**Table 10:** MANOVA tests´results.

	*Df*	*Pillai*	*approx F*	*num Df*	*den Df*	*Pr(>F)*
**Forelimb Proximal Phalanx**						
Subspecies	3	1.3329	1.3993	36	63	0.1204
Habitat	2	1.1725	2.4794	24	42	**0.0048**
Mobility	2	0.9322	1.5279	24	42	0.1124
ssp+habitat+mobility	6	3.1578	1.9443	72	126	**0.0005**
Subspecies*Habitat	5	2.7339	2.1113	60	105	**0.0004**
Subspecies*Mobility	5	2.4937	1.7412	60	105	**0.0065**
Mobility*Habitat	3	1.5963	1.9901	36	63	**0.0083**
**Hindlimb Proximal Phalanx**						
Subspecies	3	1.5507	2.2165	42	87	**0.0009**
Habitat	2	1.2061	3.1468	28	58	**0.0001**
Mobility	2	1.1497	2.8006	28	58	**0.0004**
ssp+habitat+mobility	6	3.0831	2.1894	84	174	**7.503e-06**
Subspecies*Habitat	5	2.7631	2.5588	70	145	**9.987e-07**
Subspecies*Mobility	5	2.7067	2.4449	70	145	**3.094e-06**
Habitat* Mobility	3	1.526	2.1446	42	87	**0.0014**

MANOVA tests´results for forelimb and hindlimb proximal phalanges according to subspecies (*R*.*t*. *caribou*, *R*.*t*. *granti*, *R*.*t*. *groenlandicus* and *R*.*t*. *pearyi*), habitat (mountain, tundra and boreal forest) and mobility type (sedentary, planarly and altitudinal) and their interactions. A significant contribution was considered for *P* value <0.05 (**in bold**).

**Table 11:** MANOVA pairwise comparisons results.

	*Pillai*	*approx F*	*num DF*	*den DF*	*Pr(>F)*
*Rtcaribou_BorealForest_Sedentary—Rtcaribou_Mountain_Altitudinal*	0.68108	2.8475	12	16	0.55760
*Rtcaribou_BorealForest_Sedentary—Rtcaribou_Mountain_Sedentary*	0.75484	4.1054	12	16	0.10423
*Rtcaribou_BorealForest_Sedentary—Rtcaribou_Tundra_Planarly*	0.81225	5.7684	12	16	**0.01683**
*Rtcaribou_BorealForest_Sedentary—Rtgranti_Tundra_Planarly*	0.61398	2.1207	12	16	1.00000
*Rtcaribou_BorealForest_Sedentary—Rtgroenlandicus_Tundra_Planarly*	0.60321	2.0270	12	16	1.00000
*Rtcaribou_BorealForest_Sedentary—Rtpeary_Tundra_Planarly*	0.80121	5.3740	12	16	**0.02508**
*Rtcaribou_Mountain_Altitudinal—Rtcaribou_Mountain_Sedentary*	0.48980	1.2800	12	16	1.00000
*Rtcaribou_Mountain_Altitudinal—Rtcaribou_Tundra_Planarly*	0.74740	3.9450	12	16	0.12702
*Rtcaribou_Mountain_Altitudinal—Rtgranti_Tundra_Planarly*	0.40312	0.9005	12	16	1.00000
*Rtcaribou_Mountain_Altitudinal—Rtgroenlandicus_Tundra_Planarly*	0.40754	0.9172	12	16	1.00000
*Rtcaribou_Mountain_Altitudinal—Rtpeary_Tundra_Planarly*	0.63921	2.3622	12	16	1.00000
*Rtcaribou_Mountain_Sedentary—Rtcaribou_Tundra_Planarly*	0.65022	2.4786	12	16	0.96660
*Rtcaribou_Mountain_Sedentary—Rtgranti_Tundra_Planarly*	0.54285	1.5833	12	16	1.00000
*Rtcaribou_Mountain_Sedentary—Rtgroenlandicus_Tundra_Planarly*	0.41165	0.9329	12	16	1.00000
*Rtcaribou_Mountain_Sedentary—Rtpeary_Tundra_Planarly*	0.51080	1.3922	12	16	1.00000
*Rtcaribou_Tundra_Planarly—Rtgranti_Tundra_Planarly*	0.75086	4.0185	12	16	0.11596
*Rtcaribou_Tundra_Planarly—Rtgroenlandicus_Tundra_Planarly*	0.52656	1.4829	12	16	1.00000
*Rtcaribou_Tundra_Planarly—Rtpeary_Tundra_Planarly*	0.57170	1.7798	12	16	1.00000
*Rtgranti_Tundra_Planarly—Rtgroenlandicus_Tundra_Planarly*	0.58240	1.8595	12	16	1.00000
*Rtgranti_Tundra_Planarly—Rtpeary_Tundra_Planarly*	0.68400	2.8861	12	16	0.52725
*Rtgroenlandicus_Tundra_Planarly—Rtpeary_Tundra_Planarly*	0.47736	1.2178	12	16	1.00000

Results of the MANOVA pairwise comparisons for forelimb proximal phalanx according to the different categories. A significant contribution was considered for *P* value <0.05 (**in bold**).

CVA results for classification (Table 12) obtained 82.35% overall accuracy according to habitat and a lower percentage in accuracy when it was performed according to mobility pattern (73.52%). These percentages are a bit higher when calculated according to 2 mobility groups (migratory/sedentary), 82.3%. Thus, classification accuracy in this case improves when mobility patterns are grouped into two categories rather than multiple categories. In all cases, Kappa statistic (see S1 File) was higher than 0.6, which indicates a substantial agreement beyond chance.

**Table 12:** Canonical Variate Analysis (CVA) results.

	PCs	CCV (%)	Kappa
**Forelimb Proximal Phalanx**			
Habitat	6	82.35	0.72
Mobility	7	73.52	0.59
**Hindlimb Proximal Phalanx**			
Habitat	11	84.09	0.75
Mobility	12	86.36	0.79

Canonical Variate Analysis (CVA) indicating the number of PC scores (PCs) used in the analysis, the percentage of correct cross-validated classification according to habitat (mountain, tundra and boreal forest) and mobility type (sedentary, planarly and altitudinal) for fore and hindlimb phalanges.

### Hindlimb phalanges

**Fig 7:**
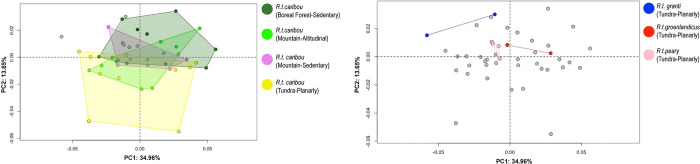
Principal component analysis. Scatter plots of the two first axes (PC1 and PC2) of principal component analyses performed on the shape data (hindlimb phalanx) according to subspecies (*R*.*t*. *caribou*, *R*.*t*. *granti*, *R*.*t*. *groenlandicus* and *R*.*t*. *pearyi*), habitat (mountain, tundra and boreal forest) and mobility type (sedentary, planarly and altitudinal). The result of the same PCA is expressed in two scatterplots (for a better visualization). On the left, the highlighted individuals correspond to *R*.*t*. *caribou* subspecies, and on the right, those of the remaining subspecies.

First two PCs accounts for 48.61% of the morphological variance (Fig 7). Rtcaribou_BForest_Sed are distributed towards positive and negative values of PC1, while Rtcaribou_Mount_Sed fell towards positive values of PC1 and Rtcaribou_Mount_Alt towards negative values of PC2. *R*.*t*. *peary* as well as *R*.*t*. *granti* and *groenlandicus*, fall mainly towards positive values of PC2, whereas Rtcaribou_Tundra_Plan were falling towards both positive and negative values of PC1.

**Fig 8:**
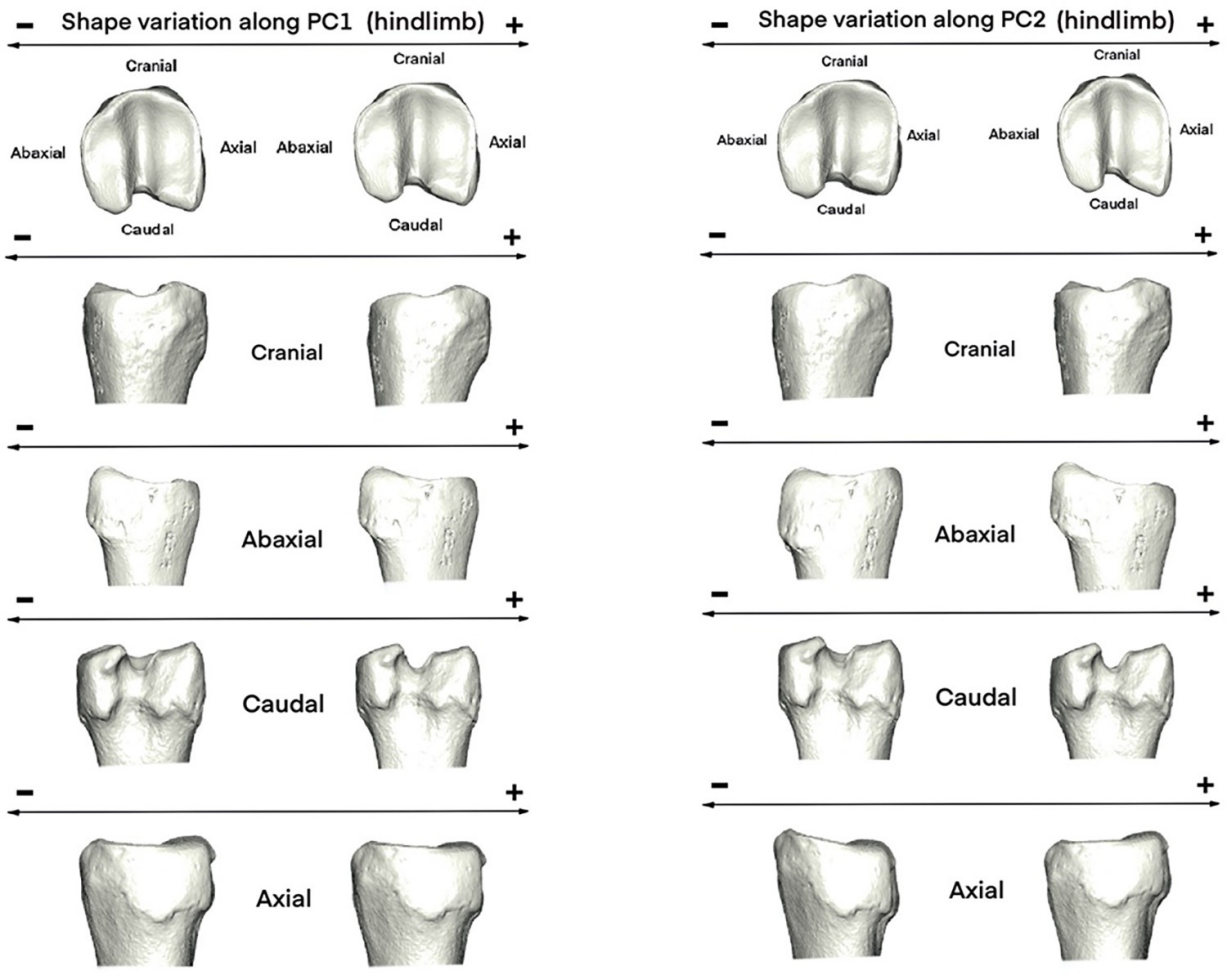
Visualization of shape variation. Shape variation visualized by deforming the mean shape along negative and positive PC1 and PC2 values (magnified by a scale factor of 0.1). Hindlimb phalanges.

The PC1 minimum configuration (Fig 8) seemed to be more stretched craniodorsally and narrower mediolaterally. Contrary, towards the positive values of PC1 or maximum configuration expressed a theoretical morphology that was broader mediolaterally and more elongated dorsally in the lateral articular surface.

Towards positive values of PC1 (Fig 8), the morphology tends to be more curved towards the abaxial side. On this same side, the configuration is more massive towards positive than negative values, however in the caudal and axial views the morphology is wider and more pronounced towards negative than positive values of the PC1 axis.

In PC2 (Fig 8), the morphological configuration in this axis stands out for being more pronounced towards its positive values. The abaxial view shows a greater protuberance in the lower part of the articular surfaces towards the positive values of the PC2 axis and a flatter configuration towards the negative ones. However, in axial view, it tends to bulge more towards negative values, while being flatter towards positive values of the axis.

Regarding PC2 (Fig 8), we find the opposite as in PC1. The theoretical morphology at the minimum appeared to be more massive mediolaterally in the proximal articular surface and with a deeper groove. However, it was narrower abaxially at the PC2 maximum.

MANOVA analyses yielded significant differences (Table 10) in shape across all categories: Subspecies (p = 0.0009); Habitat (p = 0.0001); Mobility (0.0004); Ssp+Habitat+Mobility (p = 7.503e-06); Subspecies*Habitat (p = 9.987e-07); Subspecies*Mobility (p = 3.094e-06); Mobility*Habitat (p = 0.0014). Multiple comparisons (Table 13) according to Ssp+Habitat+Mobility yielded significant results between Rtcaribou_BForest_Sed on one hand, Rtgranti and Rtpeary_ Tundra_ Planarly on the other (p = 0.012 and 0.004 respectively); Rtcaribou_Mount_Alt and Rtcaribou and Rtgranti_Tundra_Plan (p = 0.039 and 0.034) and finally between Rtcaribou_Tundra_Plan and Rtgranti and Rtpeary_Tundra_Plan (p = 0.005 and 0.03).

**Table 13:** MANOVA pairwise comparisons results.

	*Pillai*	*approx F*	*num DF*	*den DF*	*Pr(>F)*
*Rtcaribou_BorealForest_Sedentary—Rtcaribou_Mountain_Altitudinal*	0.65869	3.3084	14	24	0.102651
*Rtcaribou_BorealForest_Sedentary—Rtcaribou_Mountain_Sedentary*	0.51214	1.7996	14	24	1.000000
*Rtcaribou_BorealForest_Sedentary—Rtcaribou_Tundra_Planarly*	0.64209	3.0754	14	24	0.158975
*Rtcaribou_BorealForest_Sedentary—Rtgranti_Tundra_Planarly*	0.72532	4.5268	14	24	**0.012574**
*Rtcaribou_BorealForest_Sedentary—Rtgroenlandicus_Tundra_Planarly*	0.59728	2.5425	14	24	0.451379
*Rtcaribou_BorealForest_Sedentary—Rtpeary_Tundra_Planarly*	0.75467	5.2734	14	24	**0.004002**
*Rtcaribou_Mountain_Altitudinal—Rtcaribou_Mountain_Sedentary*	0.36959	1.0050	14	24	1.000000
*Rtcaribou_Mountain_Altitudinal—Rtcaribou_Tundra_Planarly*	0.69112	3.8358	14	24	**0.039837**
*Rtcaribou_Mountain_Altitudinal—Rtgranti_Tundra_Planarly*	0.69538	3.9134	14	24	**0.034828**
*Rtcaribou_Mountain_Altitudinal—Rtgroenlandicus_Tundra_Planarly*	0.48744	1.6303	14	24	1.000000
*Rtcaribou_Mountain_Altitudinal—Rtpeary_Tundra_Planarly*	0.62676	2.8786	14	24	0.232115
*Rtcaribou_Mountain_Sedentary—Rtcaribou_Tundra_Planarly*	0.47110	1.5269	14	24	1.000000
*Rtcaribou_Mountain_Sedentary—Rtgranti_Tundra_Planarly*	0.59613	2.5304	14	24	0.462520
*Rtcaribou_Mountain_Sedentary—Rtgroenlandicus_Tundra_Planarly*	0.45291	1.4192	14	24	1.000000
*Rtcaribou_Mountain_Sedentary—Rtpeary_Tundra_Planarly*	0.45560	1.4347	14	24	1.000000
*Rtcaribou_Tundra_Planarly—Rtgranti_Tundra_Planarly*	0.74778	5.0826	14	24	**0.005313**
*Rtcaribou_Tundra_Planarly—Rtgroenlandicus_Tundra_Planarly*	0.53954	2.0087	14	24	1.000000
*Rtcaribou_Tundra_Planarly—Rtpeary_Tundra_Planarly*	0.69918	3.9845	14	24	**0.030831**
*Rtgranti_Tundra_Planarly—Rtgroenlandicus_Tundra_Planarly*	0.67240	3.5185	14	24	0.069901
*Rtgranti_Tundra_Planarly—Rtpeary_Tundra_Planarly*	0.44625	1.3815	14	24	1.000000
*Rtgroenlandicus_Tundra_Planarly—Rtpeary_Tundra_Planarly*	0.54770	2.0759	14	24	1.000000

Results of the MANOVA pairwise comparisons for hindlimb proximal phalanx according to the different categories. A significant contribution was considered for *P* value <0.05 (**in bold**).

Regarding CVA cross-validated results (Table 12), accuracy values obtained were over 80%. According to habitat (mountain, tundra, boreal forest), the overall accuracy was 84.09%, and 86.36% for mobility pattern (planarly, altitudinal and sedentary). In all cases Kappa metric was higher than 0.79 (see S1 File), what means a good agreement between predicted and true labels.

### Shape variation amongst *Rangifer tarandus caribou*

**Fig 9:**
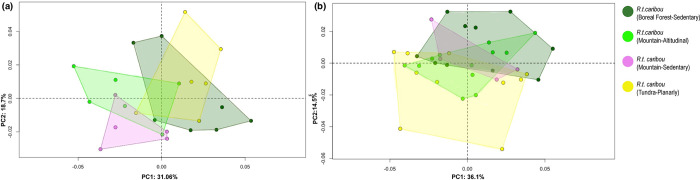
Principal component analysis. Scatter plots of the two first axes (PC1 and PC2) of principal component analyses performed on the shape data for *Rangifer tarandus caribou* according to habitat (mountain, tundra and boreal forest) and mobility type (sedentary, planarly and altitudinal); (a) Forelimb; (b) hindlimb.

In forelimb phalanges, the first two PCs account for 49.76% of the total variance (Fig 9A). Both Altitudinal and Sedendary Rtcaribou_Mountain are distributed in the morphospace towards positive and negative values of PC2, while Rtcaribou_BForest are distributed mainly towards positive values of PC1 and Rtcaribou_Tundra_Planarly along positive values of PC2. Positive PC1 values reflect the characteristics of Rtcaribou_BForest_Sedentary individuals, which means a more prominent axial articular surface cranially compared to the negative PC1 values.

**Fig 10:**
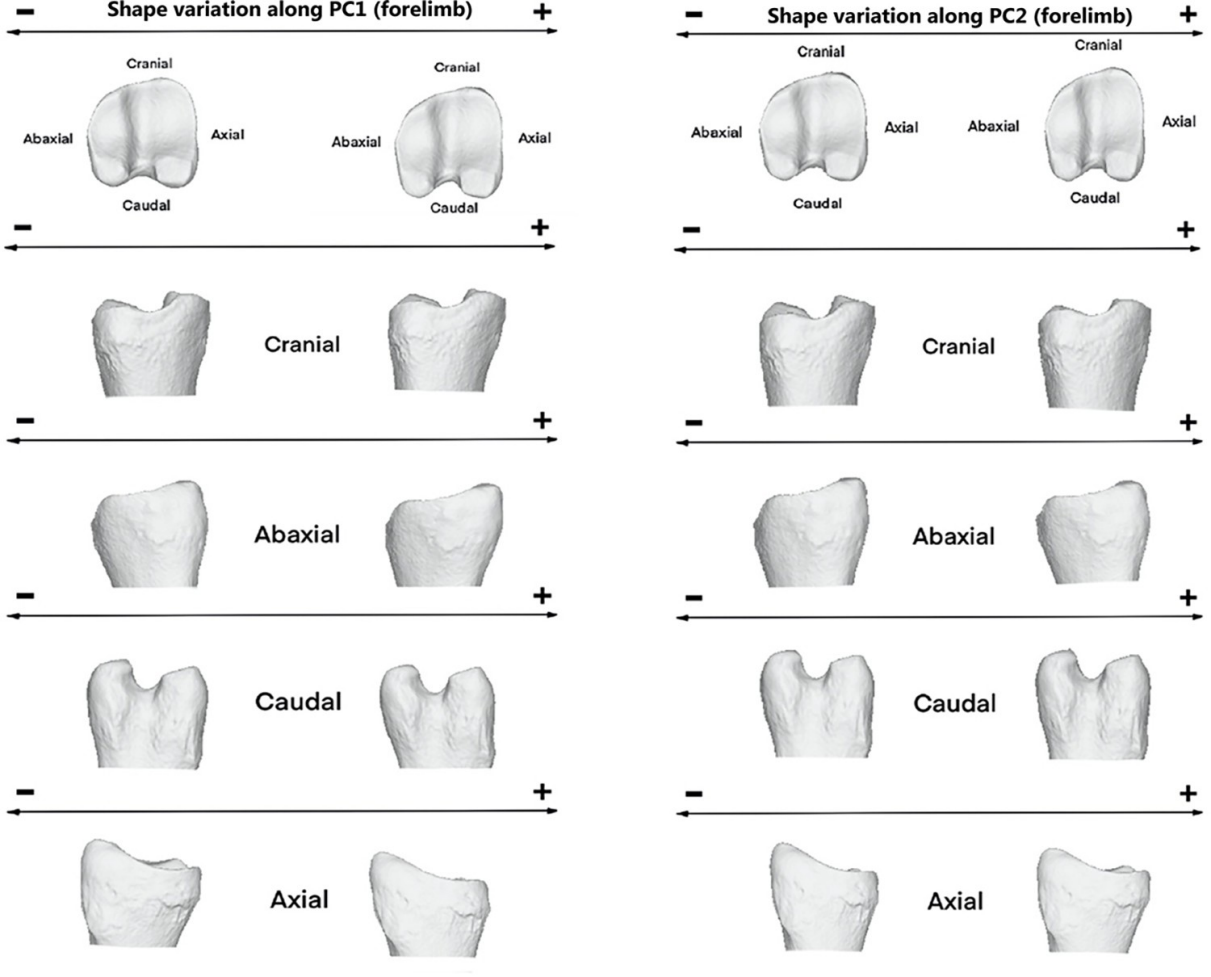
Visualization of shape variation. Shape variation visualized by deforming the mean shape along negative and positive PC1 and PC2 values (magnified by a scale factor of 0.1). Forelimb phalanges from *R*.*t*. *caribou*.

Individuals with positive PC2 values (sedentary specimens) display a joint morphology (Fig 10) that is more stretched abaxially and elongated in the craniocaudal axis; however, individuals with negative PC2 values have a joint that is wider in the abaxial axis (Rtcaribou_Tundra_Planarly and Rtcaribou_Mountain_Altitudinal).

In the case of hindlimb phalanges, the analysis of the PCA reveals a complex configuration. PC1 and PC2 account for 50% of the variance (Fig 9B). Rtcaribou_tundra and Mountain are mainly distributed along PC1 with positive and negative values. In contrast, specimens of Rtcaribou_BForest_Sedentary are mainly distributed along positive values of PC1 and PC2, overlapping with some Rtcaribou_Mountain_Altitudinal individuals.

**Fig 11:**
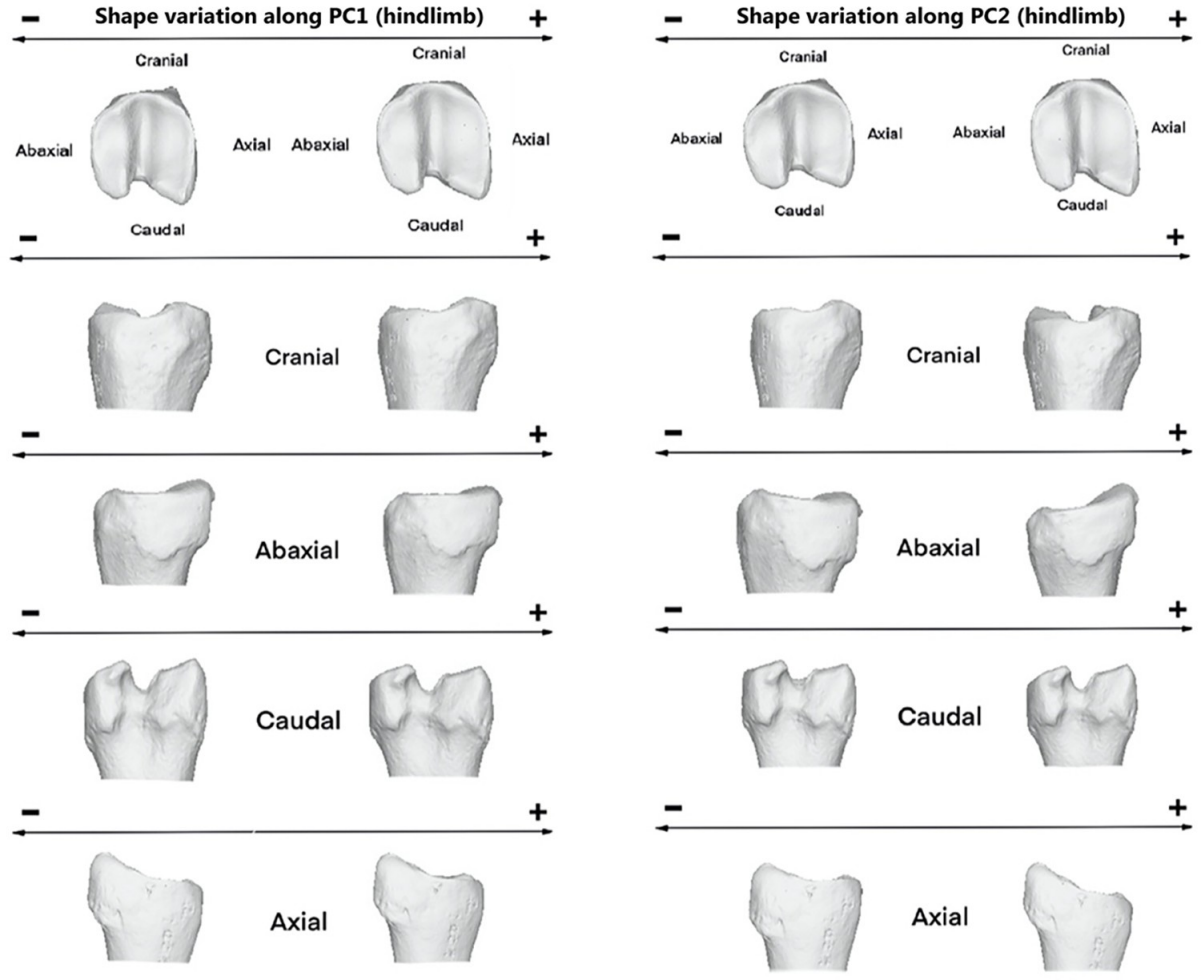
Visualization of shape variation. Shape variation visualized by deforming the mean shape along negative and positive PC1 and PC2 values (magnified by a scale factor of 0.1). Hindlimb phalanges from *R*.*t*. *caribou*.

Thus, specimens toward positive values of PC1 are shown as having an axial articular surface that is more elongated than those towards negative values (Fig 11). Those specimens towards PC2 positive values show a stretched morphology on the abaxial axis and a more elongated axial surface towards the caudal side, whereas those that have negative values have the opposite configuration (wider abaxially and narrower craniocaudally).

In both fore- and hindlimb phalanges (Table 14), there are significant differences (p<0.05) according to habitat, mobility, and the interactions between the two. Nonetheless, pairwise comparisons revealed differences between all habitat categories and between altitudinal and planarly migration types in forelimb phalanx (Tables 15 and 16).

**Table 14:** MANOVA tests´results.

	*Df*	*Pillai*	*approx F*	*Num Df*	*den Df*	*Pr(>F)*
**Forelimb Proximal Phalanx**						
Habitat	2	1.4987	4.6508	18	28	**0.000148**
Mobility	2	1.2746	2.7334	18	28	**0.008286**
Habitat* Mobility	3	1.8801	2.6115	27	42	**0.002546**
**Hindlimb Proximal Phalanx**						
Habitat	2	1.3199	3.7197	24	46	**6.441e-05**
Mobility	2	1.1897	2.814	24	46	**0.001268**
Habitat* Mobility	3	1.6198	2.2494	36	69	**0.001934**

MANOVA tests´results for forelimb and hindlimb proximal phalanges for *Rangifer tarandus caribou* according to habitat (mountain, tundra and boreal forest) and mobility pattern (sedentary, planarly and altitudinal) and their interaction. A significant contribution was considered for *P* value <0.05 (**in bold**).

**Table 15:** MANOVA pairwise comparisons results (habitat-forelimb phalanx).

Habitat	*Pillai*	*approx F*	*num DF*	*den DF*	*Pr(>F)*
*BorealForest—Mountain*	0.72827	3.8712	9	13	**0.041324**
*BorealForest—Tundra*	0.73151	3.9354	9	13	**0.038709**
*Mountain—Tundra*	0.80401	5.9257	9	13	**0.006563**

Results of the MANOVA pairwise comparisons for *Rangifer tarandus caribou* (forelimb phalanx) according to habitat type (mountain, tundra, boreal forest). A significant contribution was considered for *P* value <0.05 (**in bold**).

**Table 16:** MANOVA pairwise comparisons results (mobility-forelimb phalanx).

Mobility	*Pillai*	*approx F*	*num DF*	*den DF*	*Pr(>F)*
*Altitudinal—Planarly*	0.73395	3.9849	9	13	**0.03682**
*Altitudinal—Sedentary*	0.49471	1.4142	9	13	0.82844
*Planarly—Sedentary*	0.76317	4.6545	9	13	**0.01933**

Results of the MANOVA pairwise comparisons for *Rangifer tarandus caribou* (forelimb phalanx) according to mobility pattern (sedentary, planarly, altitudinal). A significant contribution was considered for *P* value <0.05 (**in bold**).

All habitat categories (Table 17) and mobility (Table 18) only for Altitudinal-Planarly showed significant differences for hindlimb phalanges. Performance according to Habitat+Mobility (Table 19) yielded differences between Rtcaribou_BForest_Sedendary and Rtcaribou_Mountain_Altitudinal (p = 0.01), BForest_Sedentary and Tundra_Planarly (p = 0.02) and Mountain_Altitudinal and Tundra_Planarly (p = 0.03).

**Table 17:** MANOVA pairwise comparisons results (habitat-hindlimb phalanx).

Habitat	*Pillai*	*approx F*	*num DF*	*den DF*	*Pr(>F)*
*BorealForest—Mountain*	0.69207	4.1204	12	22	**0.005958**
*BorealForest—Tundra*	0.67136	3.7452	12	22	**0.010688**
*Mountain—Tundra*	0.62338	3.0345	12	22	**0.034812**

Results of the MANOVA pairwise comparisons for *Rangifer tarandus caribou* (hindlimb phalanx) according to habitat type (mountain, tundra, boreal forest). A significant contribution was considered for *P* value <0.05 (**in bold**).

**Table 18:** MANOVA pairwise comparisons results (mobility-hindlimb phalanx).

Mobility	*Pillai*	*approx F*	*num DF*	*den DF*	*Pr(>F)*
*Altitudinal—Planarly*	0.67123	3.7430	12	22	**0.01072**
*Altitudinal—Sedentary*	0.57331	2.4633	12	22	0.09655
*Planarly—Sedentary*	0.57472	2.4776	12	22	0.09405

Results of the MANOVA pairwise comparisons for *Rangifer tarandus caribou* (hindlimb phalanx) according to mobility pattern (sedentary, planarly, altitudinal). A significant contribution was considered for *P* value <0.05 (**in bold**).

**Table 19:** MANOVA pairwise comparisons results (considering all the categories).

	*Pillai*	*approx F*	*num DF*	*den DF*	*Pr(>F)*
*Rtcaribou_BorealForest_Sedentary—Rtcaribou_Mountain_Altitudinal*	0.68956	3.8871	12	21	**0.01932**
*Rtcaribou_BorealForest_Sedentary—Rtcaribou_Mountain_Sedentary*	0.52888	1.9645	12	21	0.50795
*Rtcaribou_BorealForest_Sedentary—Rtcaribou_Tundra_Planarly*	0.67718	3.6709	12	21	**0.02695**
*Rtcaribou_Mountain_Altitudinal—Rtcaribou_Mountain_Sedentary*	0.34718	0.9307	12	21	1.00000
*Rtcaribou_Mountain_Altitudinal—Rtcaribou_Tundra_Planarly*	0.67132	3.5743	12	21	**0.03136**
*Rtcaribou_Mountain_Sedentary—Rtcaribou_Tundra_Planarly*	0.37552	1.0523	12	21	1.00000

Results of the MANOVA pairwise comparisons for hindlimb proximal phalanx according to the different categories. A significant contribution was considered for *P* value <0.05 (**in bold**).

### Sexual dimorphism on size (*R*.*t*. *caribou*)

Despite the fact that sex is frequently considered an important variable in reindeer morphological studies [75, 77, 83, 88] size and shape sexual dimorphism can hardly be addressed in our sample, due to the low number of specimens of known sex (Table 2). Only the *R*. *t*. *caribou* sample have specimens of both sexes. The lack of enough specimens prevents further analyses due to low statistical power to detect meaningful differences between groups. In contrast, boxplots were used to visualize log-centroid size distribution. Thus, when the variation in log-transformed centroid size is displayed in boxplots (see S2 File) according to male/female it can be observed that males tend to be larger in size than females, although there is an important overlap. Considering these results and our limited sample in terms of specimens of known sex, we discarded the sex variable for further analyses.

### Allometry

For forelimb phalanx, the percentage of variance related to size was low, only a 5.90% and the allometry was significant (p = 0.04). However, for hindlimb phalanx, the percentage of variance related to shape was even lower, at 2.34%, and allometry was not significant (p = 0.38).

**Fig 12:**
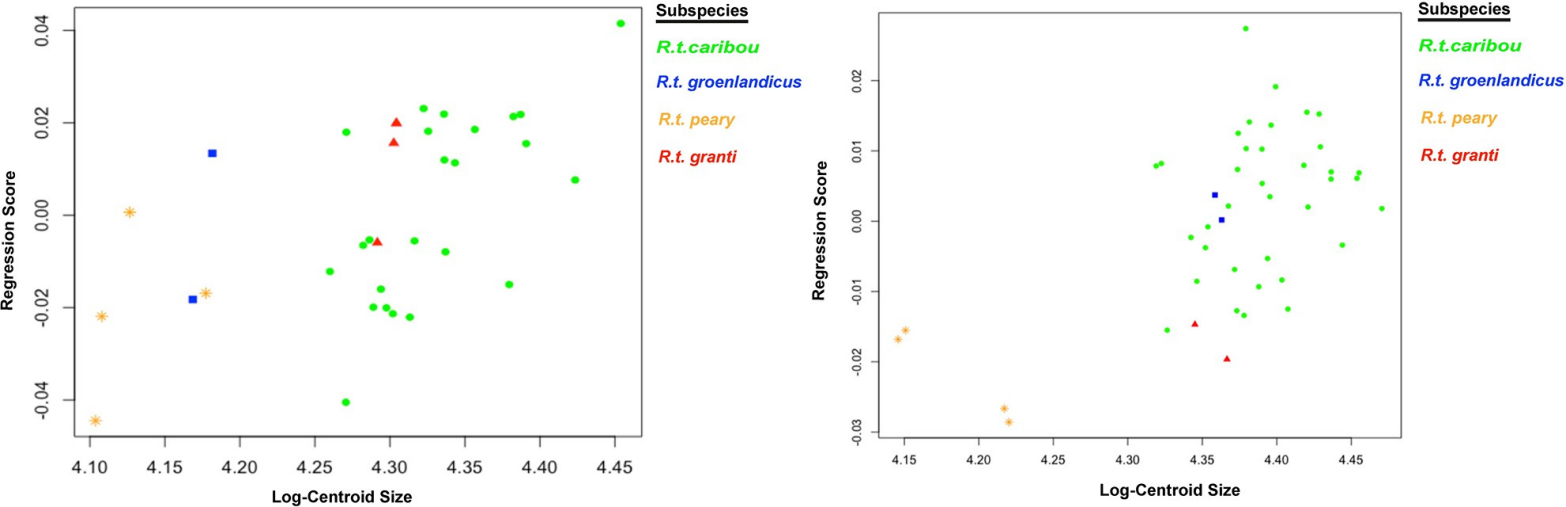
Multivariate regression plots. Multivariate regression plots performed on shape data (regression scores) and log-transformed centroid size (Log-Centroid Size) according to subspecies (*R*.*t*. *caribou*, *R*.*t*. *granti*, *R*.*t*. *groenlandicus* and *R*.*t*. *pearyi*); Forelimb (left) and hindlimb (right) phalanges.

Multivariate regression (Fig 12) of shape scores against log-transformed centroid size showed a clear separation of *R*. *t*. *caribou* with the rest of the subspecies (except *R*. *t*. *granti*) in forelimb phalanx. For hindlimb phalanx (Fig 12), *R*. *t*. *peary* is completely separated from the rest of the subspecies, which overlap among them.

## Discussion

In the present study, we have examined the morphology of caribou proximal phalanx and its relationship with mobility and habitat type in order to potentially apply into archaeological record for palaeoecological reconstructions. Despite the fact that a wider and more complete sample for further analyses is needed, this work represents a first step in this regard.

As we have already mentioned along our study, the main limitations of our sample are the size and the lack of information on the sex of the specimens. Due to the fact that sedentary populations are classified as “Endangered”, the acquisition of samples from these herds was more difficult (since they are barely extant in museums, it was only possible to acquire them through samples sent by biologists acquired when a caribou died in a traffic accident or was found dead in the forest), resulting in uneven sample sizes. Regarding sex, most of the specimens from museum collections were collected at the end of the 19th or beginning of the 20th century and they did not specify the sex, so we can only know it from those that were collected in situ by biologists nowadays. Because only few individuals within the sample population are of known sex, we cannot directly evaluate the relationship between tested values and sex regarding to habitat or mobility.

Specimens with bone pathologies [71, 72] were excluded from the study to avoid this bias in our sample. Despite the limitations imposed by our sample size, there is a consonance between the statistical analyses and the results in the configuration of the morphospace. Due to this reason, we considered including the subspecies category as a preliminary step while waiting for a larger sample size.

Studying joint surfaces allows for the use of complete or fragmented bones, increasing the sample size that can be used in archaeological records [67]. Furthermore, when compared to previous studies with linear measurements on reindeer phalanges, a 3D GMM study has the advantage of allowing a deeper study in the morphology of these phalanges, and is able to accurately capture shape variation, even with a small sample size (e.g. altitudinal migration was difficult to explore using linear measurements, cf. [41]). For validation of the methods presented here, we recommend similar studies on caribou populations in other regions so that we can assess the preliminary variations we observed.

### Interpreting size variation: A complex interplay of sexual dimorphism and migration costs?

Size variations are mainly imputable to subspecies differences. *R*. *t*. *caribou* are statistically significantly larger than *R*. *t*. *pearyi*, which was expected, since the latter is amongst the smallest *R*. *t*. subspecies [119]. Peary Caribou, inhabit the Canadian Arctic Archipelago (island syndrome) and northern of the Boothia Peninsula [94, 119]. They are adapted to the Arctic environment having a compact body size for conserving heat (Allen´s rule) [120] and to the limited plant growth with a highly compressed growing season and long periods of snow-covered frozen standing vegetation [119]. All these facts explain their lower centroid sizes.

At an intra-subspecies scale, size differences are not significant between populations of *R*.*t*. *caribou*. Forelimb phalanges of specimens that perform altitudinal migration tend to be slightly larger than those belonging to planarly or sedentary migratory behaviour, but it is yet unknown if this is linked to real population differences or unbalanced sex ratios in our sample.

If confirmed, these size differences might be related to the migration costs. Migration of long distances might increase access to high quality food or reduce the risk of predation but it also involves an energetic cost that may be reflected in body conditions of animals [121, 122]. The proportional cost of horizontal or planarly travel that tundra caribou performs increases with decreasing body size and long movements, particularly in winter, have a negative impact on caribou body size [123, 124]. However, at present, there is no evidence of a statistically significant influence of habitat or mobility on phalanx size.

Size differences tend to be more pronounced on forelimb than hindlimb members. This could be explained by the fact that forelimb supports more stress as they carry greater share of the body weight [75–77, 125–128]. The use of forelimb more actively is also linked to the search of food, breaking through ice layers in many cases [69, 70, 75]. It is, however, important to keep in mind that these results do not satisfy the thresholds of statistical significance, and therefore should be interpreted carefully.

### Shape variation: The impact of subspecies, substrate type and snow cover?

Despite the fact that significant shape differences exist between populations, it is difficult to identify a specific morphology that corresponds to a given habitat or mobility type.

If we consider CVA, in terms of classification instead of associated morphological description, CVA is specifically designed to identify linear combinations of variables that best discriminate between groups. It maximizes the separation between groups while minimizing the variation within groups, and thus can be more effective in identifying the underlying differences between groups. For forelimb phalanges, the results suggest that habitat may be a stronger predictor of classification accuracy than mobility pattern. However, on hindlimb phalanges, these ones indicate that both habitat type and mobility pattern are important predictors of the observed patterns.

Thus, these outcomes suggest that the morphology of caribou forelimb phalanges is more heavily influenced by the type of habitat in which they inhabit than their mobility pattern. This could mean that the forelimbs are adapted for specific types of terrain (e.g. rocky and mountainous terrain). In contrast, the results for the hindlimb phalanges indicate that both habitat type and mobility pattern are significant predictors of the observed patterns, suggesting that they are better suited for more dynamic activities (e.g. running). Thus, the different predictors of classification accuracy between the two limb sets likely reflect the different functional roles of each set of phalanges. The results are consistent with the MANOVA results, which also indicate that habitat variable plays an important role in the configuration of the shape of the phalanx morphology.

Results on forelimb phalanx revealed, along PC1, on one hand, a joint with a more massive morphology towards cranial side and medio-laterally narrower (mainly present in Rtcaribou_BForest_Sed and partially on Rtcaribou_Tundra_Plan), and on the other hand, proximal joints that appeared to be more pronounced mediolaterally rather than craniocaudally (mostly observed in mountain *R*.*t*. *caribou* and *R*.*t*. *granti*, *R*.*t*. *groenlandicus* and *R*.*t*. *peary*). There is however no clear distinction according to habitat or mobility. In spite of the difficulty of establishing the functional implications of these morphological differences, it could suggest adaptations to different types of movement or stresses placed on the forelimb phalanges in different environments. For example, the more massive joint towards the cranial side and medio-laterally narrower morphology observed in individuals inhabiting the boreal forest may provide greater stability when moving through snow, whereas the more pronounced mediolateral morphology observed in mountain individuals may provide greater flexibility when traversing uneven terrain.

Towards the maximum configuration of PC2 we observe a morphology that is wider in the axial-abaxial axe and narrower craniocaudally (especially for sedentary *R*.*t*. *caribou* from the mountains, and also for planarly migrating *R*.*t*. *granti* from the tundra) and the opposite configuration along negative values of PC2, with specimens that are both sedentary or migratory, inhabiting mountain, boreal forest or tundra. At this point, PC2 is playing a smaller role in determining the shape of the joint., In other words, there is no clear distinction between the morphology of populations based on their migration behaviour or habitat.

Forelimb phalanx from sedentary *R*.*t*. *caribou* inhabiting the mountains overlaps to a lesser extent in the morphospace with the other populations (especially when the *R*.*t*. *caribou* subspecies is analyzed separately) and reflects a more characteristic morphology, contrasting with the boreal forest population, which is also sedentary. The mountain caribou’s forelimb phalanx is likely to be more robust and adapted to their mountainous environment, whereas the boreal forest population’s forelimb phalanx is likely to be more adapted to the conditions found in the boreal forest (e.g. a more varied terrain, with greater amounts of wetland; a more diverse array of vegetation, including shrubs, grasses, and forbs; and a greater variety of food source). In both cases, it is worth clarifying that when we refer to sedentary populations we do so to talk about those that travel distances less than 200 km annually [see 41], so in this case substrate and snow coverage potentially have an important impact on the morphological configuration of the joint of the proximal forelimb phalanx. Our sample of boreal forest caribou comes mainly from Québec (Canada), where the forests are covered in snow more than half of the year [88]. These types of boreal forest populations also seek places with snow that can be used as refuges from predators. Sedentary montane populations, however, remain in alpine areas to feed on lichens in windswept areas where snow is absent or shallow [48]. These differences in substrate type and snow cover may also explain the slight overlap in morphological configuration between sedentary and altitudinally migrating mountain individuals: even though both inhabit mountainous ecosystems, they move in different ways (altitudinal migration vs. sedentary). While for the sedentary mountain caribou we have already discussed the characteristics of their habitat, for those that perform altitudinal migration, at the end of autumn, when the snow begins, they migrate from upper to lower elevations where the snow is shallow enough, avoiding open habitats where the snow is harder, again beginning its travel to higher elevations around February towards subalpine forests [48, 129]. This suggests that the functional morphology of this particular subspecies (Rtcaribou_Mountain_Sedentary) is well adapted to its environment, allowing to thrive in its mountainous habitat (characterized by a diverse range of elevations, and the presence of high-altitude alpine tundra, ranging from rolling hills and valleys, to steep rocky slopes, and high peaks).

Morphological differences are even harder to classify on hindlimb phalanges. All populations are distributed along PC1, and PC2 poorly distinguishes populations, indicating that the morphology of the hindlimb phalanx of each subspecies is largely dependent on the habitat and mobility pattern of the subspecies. As a result, the morphological variation of hindlimb phalanx is not so evident to interpret.

MANOVA tests showed statistically significant differences in relation to Habitat for both fore- and hindlimb phalanges, and in relation to Subspecies and Mobility for hindlimb phalanx. Shape differences were more pronounced on hindlimb than forelimb phalanges, and more marked when pooled by Habitat in both cases. This may suggest that the habitat in which *Rangifer tarandus* lives, has a significant impact on the joint shape of forelimb first phalanges, as has been proven in different Bovidae species [60, 67, 130]. To understand habitat´s influence, it is important to note the variety of environments and substrates that caribou inhabit, ranging from closed boreal forest to treeless arctic tundra passing through the mountain with a mosaic of landscapes ranging from open alpine areas to subalpine areas and low-elevation forests [69]. There is no doubt that the snow adaptation impacts the shape of the proximal joint, not only during migration, but also when the front hooves are used to dig through the snow to find lichen during snowy seasons [68].

### Shape variability at the intra-subspecies scale: The key influence of habitat?

Based on pairwise comparisons, in hindlimb phalanx, main differences on shape are found between the different subspecies, a fact that motivated the realization of the same analyses within the *Rangifer tarandus caribou* subspecies. Such intra-subspecies analysis confirmed that phalanx shape is statistically different according to Habitat and Mobility, even if only *R*.*t*. *caribou* specimens are considered.

When *R*. *t*. *caribou* was examined separately, MANOVA showed differences in habitat and mobility, as well as their interaction, in fore- and hindlimb phalanges. In pairwise comparisons, all habitat categories showed significant differences, regardless of whether they were fore- or hindleg and demonstrating (as determined by the p values) how crucial the impact of habitat on shape is. Regarding mobility, there were differences for forelimb phalanges between planarly on one hand, altitudinal and sedentary on the other, and only between altitudinal and planarly in the hindlimb ones. Considering the interaction, pairwise comparisons did not yield significantly different results in foreleg phalanx, but in hindleg, between Rtcaribou_BForest_Sed and Mountain_Altitudinal and Tundra_Planarly (respectively). Consequently, habitat has again the greatest impact on the shape, although to a lesser extent the type of mobility also has an impact. The altitudinal migration in the mountains occurs vertically, through rugged and mountainous substrates in response to snowfall conditions, forage availability, and to avoid predators. Migration in tundra occurs horizontally, with a characteristic vegetation of dwarf and upright shrubs [94, 129, 131]. These differences are not only in the substrate and the type of movement, but also in the distance traveled by the herds. Rtcaribou_Tundra_Planarly travels distances between 1,220 and 1,770 km annually, while Rtcaribou_Mountain_Altitudinal can travel between 140–240 km only in the spring migration [81, 90, 92, 131]. Furthermore, the marked difference in the hindlimb phalanx may also be explained by the implications of propulsion (along with what was previously discussed), resulting in a higher level of stress among individuals who move vertically instead of horizontally due to the substrate conditions, in addition to the fact that in quadrupeds, as it has been seen in other species, e.g. rhino, horse [126, 127, 132] the hind limbs bear relatively less weight than the fore limbs and play an additional propulsive role during locomotion.

The results show that subspecies structure the data significantly, especially for size, but habitat and mobility also have significant effects on phalanx morphology. Shape analyses on *Rangifer tarandus caribou* subspecies yielded shape differences that are especially significant in both fore and hindlimb phalanges according to habitat or mobility categories. Consequently, if the subspecies variable is excluded, we can examine how habitat has a greater impact on the morphological configuration. This can be explained by the wide variety and different habitats used by *Rangifer tarandus caribou*: those that inhabit mountain environments move among windswept alpine ridges, subalpine forests, and low-elevation forests [69]. In the Boreal Forest, in which the snow is a key feature, they seek habitats with thin and soft snow cover with a more sedentary lifestyle [88]. Those inhabiting the tundra will find many types of flowering plants, mosses, and lichens, as well as shrubs, grasses, and sedges which form swards and tussocks scattered across the tundra [94]. Thus, e.g. Rtcaribou_BForest_Sed tends to have a forelimb phalanx joint morphology with a more cranially prominent axial articular surface, compared with sedentary mountain individuals. This morphology could be linked to the adaptations that enable it to better handle the specific demands of the boreal forest environment (e.g. deep snow and the ability to dig through the snow to reach vegetation; rugged terrain and extreme cold). For example, the cranially prominent axial articular surface may allow for a greater range of motion in the joint, or it may provide additional surface area for muscle attachment, allowing for more precise and powerful movements. However, further research would be necessary to fully understand the functional implications of this morphological difference.

### Preliminary distribution on sexual dimorphism

Even though the sample size by sex is limited for both forelimb and hindlimb phalanges, there are no differences between male and female specimens from the same subspecies (*R*. *t*. *caribou*) when log-centroid sizes were displayed on boxplots to explore their distribution. In this regard, Hull [71] noted in her study to distinguish forelimb and hindlimb phalanges, that, even though differences between sexes were appreciated, they also included a large overlap, so she concluded they cannot be included in the study without additional context. The boxplots show that male phalanges are slightly larger than female ones, but they overlap. These non-significant results do not imply that these differences between the two sexes do not exist in our sample, as has been demonstrated for other anatomical parts [82], it can simply reflect that phalanges are not an indicator of this characteristic and/or a larger sample and further studies are needed.

### Allometry

Allometry is also more significant on forelimb than on hindlimb (absent in our sample) phalanges, similar to what has already been observed in other works. Previous studies on reindeer [75, 77] show that allometry was generally stronger on the forelimbs than the hindlimbs as well. It has been observed in other species as well, for example the rhinoceros [127, 128] and some bovids [130]. The fore- phalanx allometry is not very significant, but it may be compensated by the rest of the forelimbs. Thus, a predictable morphological and biomechanical response of the bone is necessary to resist the stresses caused by body mass, which is heavier (as noted above) on forelimbs due to antlers (which are heavier in males), resulting in additional forces and constraints on the foreleg bones.

## Conclusion

Reconstructing the interactions between Palaeolithic human groups and reindeer is often complicated by a general lack of data on the ethology and ecology of these past animal communities. At the end of the Late Glacial, reindeer gradually disappeared from southwestern Europe with the climate warming. As they became more fragmented geographically, different responses should be expected, such as variation in their migratory patterns. As the migration behaviours of reindeer herds during this period are largely unknown, it is difficult to interpret Late Glacial socio-economic organization and mobility strategies.

Due to the fact that locomotor morphological adaptations are closely related to habitat preferences and mobility [67, 78, 133, 134], it is possible to infer behavioural responses using an animal’s cranial and post-cranial morphology, in the case of our study, using first phalanx. Besides, phalanges are one of the most useful bones for palaeocological purposes because these elements are commonly preserved in fossil assemblages. Together with 3D geometric morphometrics, we were able to examine their morphology from an anatomical region of interest and visualize morphological variation. The present study has contributed to our understanding of the caribou proximal phalanx’s functional morphology and its relationship to mobility and habitat type (adapted to provide support on uneven terrain and harsh habitats), but it still cannot be easily applied to archaeological bone remains. A complex interplay of factors has been shown, with the impact on phalanx morphology of subspecies status, sexual dimorphism, substrate type, snow cover, migration costs, habitat, etc. This diversity of interrelated factors makes interpretation difficult with the limited sample of modern reindeer phalanges currently available. Continued efforts in the 3D scanning of phalanges should lead to further progress.

Although future studies with larger sample size are needed, this contribution represents a necessary first step to explore reindeer phalanx morphology and size differences for potential archaeological applications.

## Supporting information

S1 Table Specimens information. Detailed information related to Rangifer tarandus sample.
(DOCX)

S1 File Kappa parameter concept. Detailed description of this concept.
(DOCX)

S2 File Boxplots on log-transformed centroid size. Additional boxplots exploring size variation.
(DOCX)
