# Supplementary material for: Reconstructing past migratory behaviour of reindeer (Rangifer tarandus): Insights from geometric morphometric analysis of proximal phalanx morphology from extant caribou populations
Source: PLoS One. 2023 Aug 9;18(8):e0285487. doi: 10.1371/journal.pone.0285487 (PMC10411787; doi:10.1371/journal.pone.0285487)
Supplement: S1 Table — Detailed information related to Rangifer tarandus sample. (DOCX) [file pone.0285487.s001.docx]

**Reconstructing past migratory behaviour of reindeer (*Rangifer tarandus*): insights from geometric morphometric analysis of proximal phalanx morphology from extant caribou populations.**

**S1 Table. Specimens information:**

**S1Table**. Information related to every specimen included in the present study. Forelimb (left) and hindlimb (right) phalanges.
