# Supplementary material for: Reconstructing past migratory behaviour of reindeer (Rangifer tarandus): Insights from geometric morphometric analysis of proximal phalanx morphology from extant caribou populations
Source: PLoS One. 2023 Aug 9;18(8):e0285487. doi: 10.1371/journal.pone.0285487 (PMC10411787; doi:10.1371/journal.pone.0285487)
Supplement: S1 File — Detailed description of this concept. (DOCX) [file pone.0285487.s002.docx]

**Reconstructing past migratory behaviour of reindeer (*Rangifer tarandus*): insights from geometric morphometric analysis of proximal phalanx morphology from extant caribou populations.**

Ana Belén Galán López, Maxime Pelletier, Emmanuel Discamps.

**S1. File. Kappa parameter concept [1-3].**

The “Kappa statistic” measures accuracy by calculating the probability of a correct prediction occurring by chance alone. Kappa values range from a maximum value of 1 (perfect agreement between the model´s predictions and the true values) to -1 (rare occurrence or imperfect agreement). Thus, Kappa values of 0.3-0.6 show reasonable agreement. Values higher than these indicate a high agreement between the expected accuracy and the documented one.

1. Domínguez-Rodrigo M., Baquedano E. Distinguishing butchery cut marks from crocodile bite marks through machine learning methods. Scientific Reports. 2018; 8. doi: 10.1038/s41598-018-24071-1. pmid:29636550

2. González-Molina I, Jiménez-García B, Maíllo-Fernández J-M, Baquedano E, Domínguez-Rodrigo M. Distinguishing Discoid and Centripetal Levallois methods through machine learning. PLoS ONE. 2020; 15(12): e0244288. doi:10.1371/journal.pone.0244288

3. Galán López AB, Costamagno S, Burke A. Osteometric Study of Metapodial Bones and Phalanges as Indicators of the Behavioural Ecology of Modern Reindeer (Rangifer tarandus) and Implications for Reconstruction of Paleo Mobility. Open Quat, 2022; 8(1): p.8. DOI: <http://doi.org/10.5334/oq.106>
