# Supplementary material for: Reconstructing past migratory behaviour of reindeer (Rangifer tarandus): Insights from geometric morphometric analysis of proximal phalanx morphology from extant caribou populations
Source: PLoS One. 2023 Aug 9;18(8):e0285487. doi: 10.1371/journal.pone.0285487 (PMC10411787; doi:10.1371/journal.pone.0285487)
Supplement: S2 File — Additional boxplots exploring size variation. (DOCX) [file pone.0285487.s003.docx]

**Reconstructing past migratory behaviour of reindeer (*Rangifer tarandus*): insights from geometric morphometric analysis of proximal phalanx morphology from extant caribou populations.**

Ana Belén Galán López, Maxime Pelletier, Emmanuel Discamps.

**S2 File. Boxplots on log-transformed centroid size.**


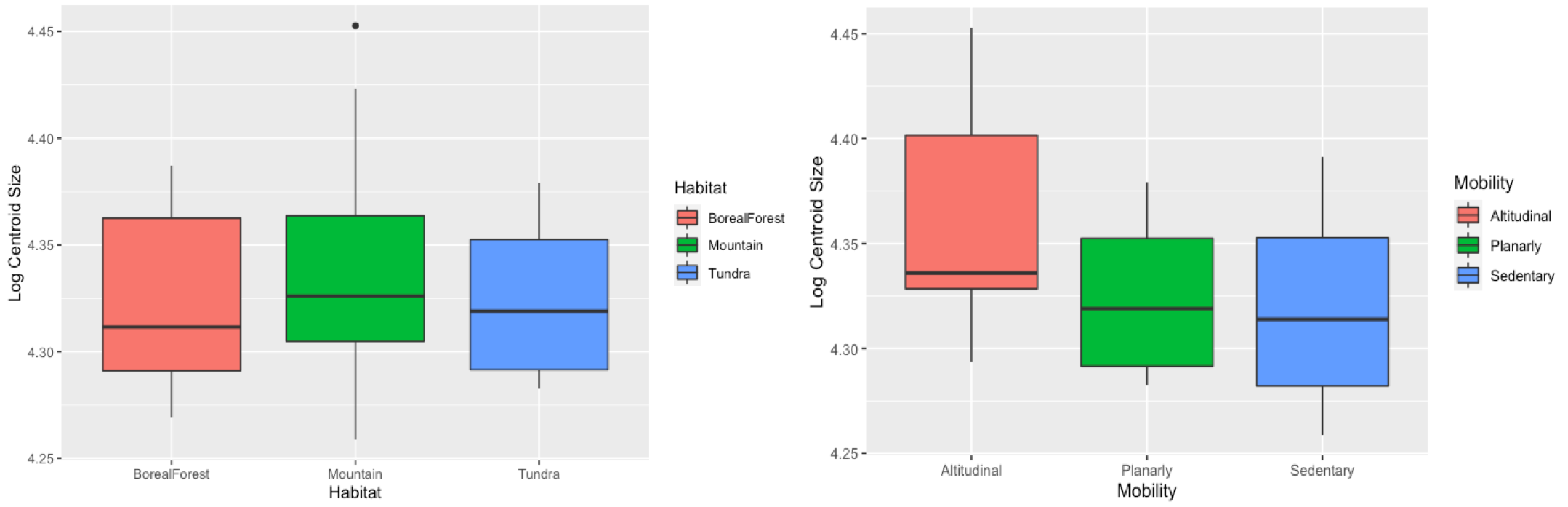


**Fig 1.** **Boxplots showing the size variation.** Log-transformed centroid size boxplot distribution on forelimb phalanges (*Rangifer tarandus caribou*) according to habitat (mountain, tundra and boreal forest) (left) and mobility type (sedentary, planarly and altitudinal) (right).


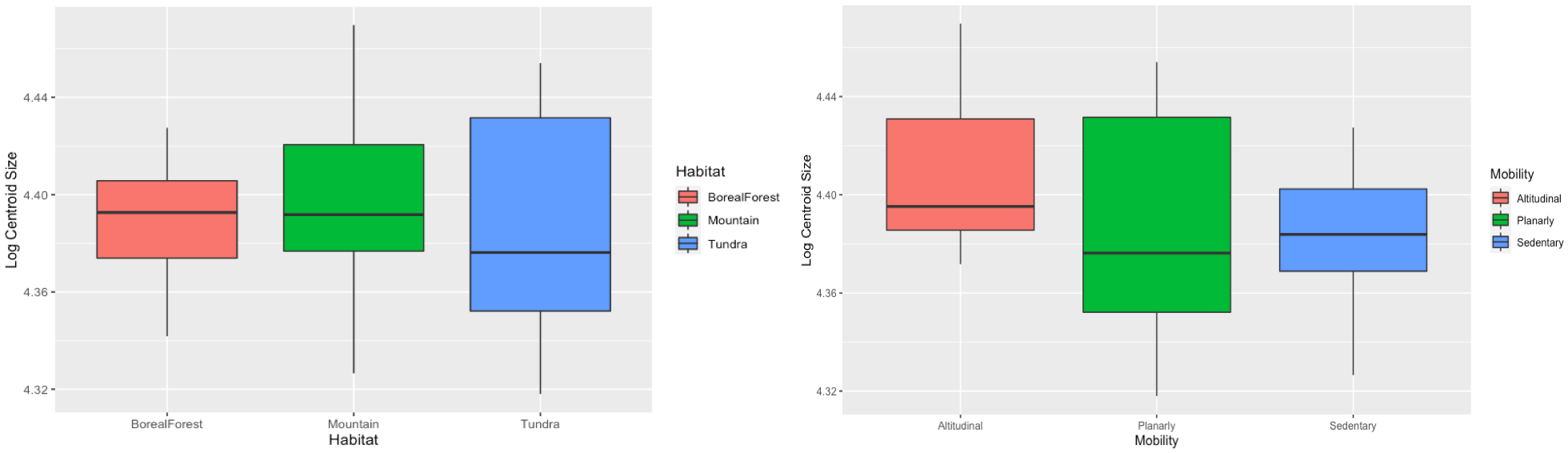


**Fig 2.** **Boxplots showing the size variation**. Log-transformed centroid size boxplot distribution on hindlimb phalanges (*Rangifer tarandus caribou*) according to habitat (mountain, tundra and boreal forest) (left) and mobility type (sedentary, planarly and altitudinal) (right).


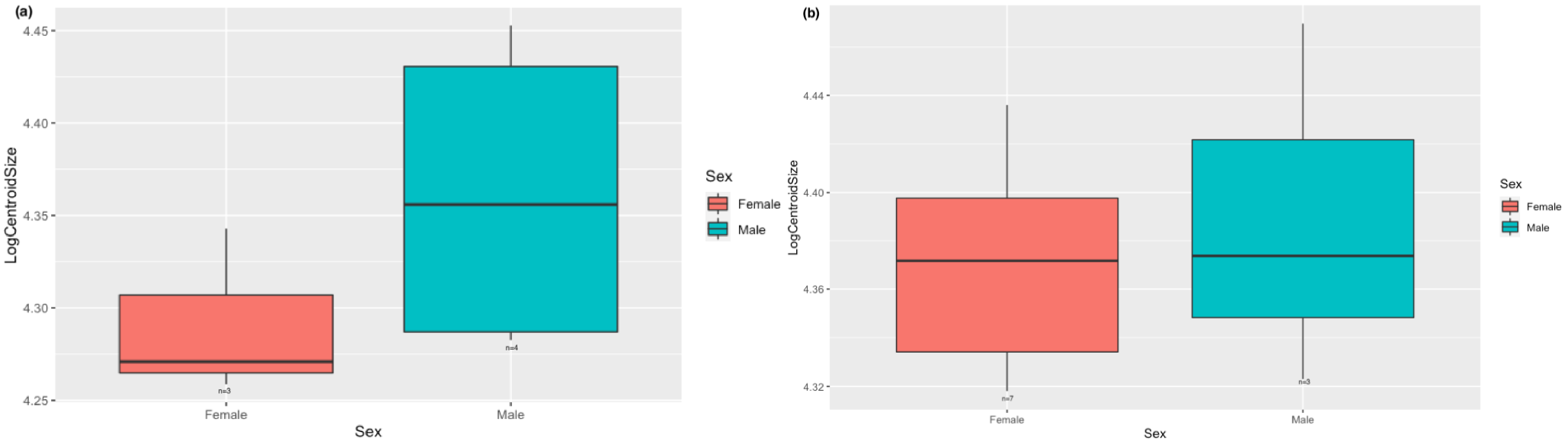


**Fig 3. Boxplots showing the size variation.** Log-transformed centroid size boxplots distribution for *Rangifer tarandus caribou* subspecies according to known sex (male-blue-and female-red-)*;* (a) Forelimb, (b) Hindlimb phalanges.
